# Patient‐Count and Perceived Workload Associations With Missed Nursing Care: A Multicenter Repeated‐Measures Study

**DOI:** 10.1111/jnu.70135

**Published:** 2026-09-08

**Authors:** Dhurata Ivziku, Daniela Tartaglini, Federica Maria Pia Ferramosca, Angelo Lanzara, Francesca Reato, Iliriana Bajrami, Marzia Lommi, Rosario Caruso

**Affiliations:** ^1^ Department of Health Professions Fondazione Policlinico Universitario Campus Bio‐Medico Rome Italy; ^2^ Faculty of Health Sciences AAB College Pristina Kosovo; ^3^ Research Unit in Nursing Science, Faculty of Medicine and Surgery Campus Bio‐Medico University Rome Italy; ^4^ Department of Anesthesia and Reanimation ASL Lecce Lecce Italy; ^5^ Bachelor's Degree Program in Nursing University of Insubria Varese Italy; ^6^ Department of Stomatology Alma Mater Europaea, Campus College Resonanca Pristina Kosovo; ^7^ Department of Biomedicine and Prevention University of Rome Tor Vergata Rome Italy; ^8^ Department of Biomedical Sciences for Health Università degli Studi di Milano Milan Italy; ^9^ Health Professions Research and Evidence Transfer Unit IRCCS MultiMedica Sesto San Giovanni Italy

**Keywords:** generalized estimating equations, medical‐surgical nursing, missed nursing care, nursing workload, patient safety, repeated‐measures research, work rhythm

## Abstract

**Introduction:**

Missed nursing care threatens quality and safety, but patient counts may not capture shift‐level demands. We examined associations of patient‐count and perceived workload indicators with missed care, separating within‐ and between‐nurse effects.

**Design:**

A multicenter observational study using repeated shift‐level measurements.

**Methods:**

The study included 502 shift records from 213 nurses in 16 medical–surgical units across six Italian hospitals; the primary analysis included 480 records from 196 nurses. Patient‐count indicators were nurse‐reported numbers of assigned, isolated, and specialist‐care patients; perceived workload included work rhythm/quantity, mental workload, emotional workload, and work organization. Grouped‐binomial generalized estimating equations modeled the proportion of applicable activities missed, with nurse clustering, robust standard errors, exchangeable correlation, and hospital fixed effects. Workloads were decomposed into within‐ and between‐nurse components, and four missed‐care domains were examined.

**Results:**

Nurses reported a mean of 6.5 missed activities per shift; 29.9% of shifts had no missed care. The seven workload indicators were jointly associated with missed care (robust Wald *χ* ^2^[7] = 22.95, *p* = 0.002). Work rhythm/quantity was the only individual indicator with a nominal *p*‐value below 0.05 (OR 1.24 per SD, 95% CI 1.03–1.49; *p* = 0.023), but it did not remain significant after Benjamini–Hochberg correction (*q* = 0.159). Assigned patient count was not clearly associated (OR 1.14, 95% CI 0.94–1.40; *p* = 0.192). In exploratory within–between analyses, the between‐nurse work rhythm/quantity component was associated with missed care (OR 1.42, 95% CI 1.11–1.82; *p* = 0.005), whereas the within‐nurse component was not (OR 1.03, 95% CI 0.90–1.17; *p* = 0.649). Domain‐specific associations did not remain significant after multiplicity adjustment.

**Conclusion:**

The workload indicators were jointly associated with missed nursing care, with secondary analyses indicating that the global signal was evident in the perceived‐workload block. However, no individual workload indicator remained statistically significant after multiplicity adjustment. Work rhythm/quantity and its between‐nurse component should therefore be regarded as exploratory signals requiring confirmation in studies with denser repeated measurements.

**Clinical Relevance:**

Workload surveillance research should evaluate patient‐count and multidimensional perceived‐workload indicators together. The present coefficient‐specific findings are insufficient to support the operational use of work rhythm/quantity as a stand‐alone workload indicator.

## 1 Introduction

Missed nursing care has become a central concept in nursing quality and patient safety research because it captures a specific, clinically meaningful form of care‐process failure: necessary nursing activities that are delayed, only partially completed, or omitted (Duman and Arikan 2026; Kalisch 2006; Kalisch, Landstrom, and Hinshaw 2009; Kalisch, Landstrom, and Williams 2009). Terminology in this field has evolved across overlapping research traditions, and labels such as “missed nursing care,” “care left undone,” and “unfinished nursing care” have often been used interchangeably despite differences in their conceptual origins and measurement approaches (Duman and Arikan 2026; Jones et al. 2015; Sarpong et al. 2026). Initially, missed nursing care was described as an “error of omission” (Kalisch 2006; Kalisch, Landstrom, and Williams 2009). The concept subsequently shifts attention from adverse events alone to the routine rationing and prioritization decisions that occur during nursing work when available resources are insufficient to meet concurrent patient needs (Kalisch et al. 2012). Conceptual and state‐of‐the‐science reviews have emphasized that missed nursing care should not be interpreted solely as an individual performance problem (Ellehave et al. 2026; Giannarou et al. 2026; Nazari et al. 2026; Sarpong et al. 2026). Rather, it is a system‐level phenomenon arising from the interaction among staffing resources, workload, organizational conditions, professional judgment, and competing care demands (Bassi et al. 2018; Giannarou et al. 2026; Jones et al. 2015).

International evidence indicates that missed nursing care is common across acute hospital settings and is associated with poorer perceived quality of care, patient safety concerns, and adverse nurse outcomes (Breno et al. 2025). In the RN4CAST study, the extent of nursing care left undone varied substantially across European hospitals and was associated with staffing levels and the quality of the nursing work environment (Ausserhofer et al. 2014). Similar findings have emerged from shift‐ and ward‐level studies in acute hospitals, in which unfinished or omitted nursing activities were associated with heavier workload, inadequate staffing, and lower ratings of care quality (Ball et al. 2014, 2016; Sarpong et al. 2026). Systematic reviews have further shown that missed nursing care is consistently related to nursing resource availability and organizational characteristics, although the magnitude and interpretation of these associations vary according to the definition of missed care, the measurement instrument used, the clinical setting, and the analytical level considered (Brera et al. 2021; Giannarou et al. 2026; Griffiths et al. 2018; Sarpong et al. 2026).

Workload is among the most frequently reported antecedents of missed nursing care, yet it remains a multidimensional and incompletely operationalized construct (Breno et al. 2025; Duman and Arikan 2026). Objective indicators, such as the number of patients assigned to each nurse or patient‐to‐nurse ratios, capture an important component of workload but do not fully represent the demands encountered during a shift (Ellehave et al. 2026). Nurses caring for similar numbers of patients may experience substantially different workloads depending on patient acuity and dependency, admissions and discharges, clinical instability, interruptions, documentation requirements, interprofessional coordination, emotional demands, and time pressure (Nazari et al. 2026). Consequently, patient count alone may underestimate the effective burden of nursing work and may only weakly discriminate between shifts that are numerically similar but operationally very different.

Recent research has therefore increasingly distinguished patient‐count workload indicators from nurses' perceived workload (Khajoei et al. 2025; Maghsoud et al. 2022). Perceived workload reflects the extent to which the demands of a shift are experienced as difficult to manage and may incorporate emotional, cognitive, temporal, and organizational pressures that are not captured by staffing ratios or patient counts (Nazari et al. 2026). Such dimensions may have independent relevance to missed nursing care because omissions result not only from the amount of work to be completed but also from interruptions, competing priorities, coordination problems, emotional strain, and limited control over workflow (Giannarou et al. 2026). Emerging evidence suggests that patient‐count and perceived workload measures may provide distinct, yet potentially complementary, information on missed care and patient experience (Bayadsi et al. 2025). Studies in specialized settings, including neonatal and intensive care units, similarly indicate that different workload assessment approaches may identify different patterns of risk and may not be interchangeable when examining missed nursing care (Fan et al. 2026; Zhong et al. 2026).

A further limitation of the existing literature concerns the level at which workload and missed nursing care are analyzed (Breno et al. 2025; Fan et al. 2026). Much of the available evidence is cross‐sectional and examines differences between nurses, wards, or hospitals (Ellehave et al. 2026; Nazari et al. 2026; Sarpong et al. 2026). These studies are essential for identifying organizational variation and structural determinants of missed care, but they provide limited insight into the dynamic nature of nursing work. Workload could vary considerably from one shift to another, even for the same nurse working in the same clinical unit (Giannarou et al. 2026). A nurse may therefore experience both relatively manageable and exceptionally demanding shifts over a short period. The association observed among nurses who are, on average, exposed to different workload levels may not be equivalent to that observed when an individual nurse experiences a temporary increase in workload relative to their usual working conditions (Fan et al. 2026).

This distinction between within‐nurse and between‐nurse variation is both analytically and practically important (Breno et al. 2025; Sarpong et al. 2026). A within‐nurse association would indicate that the same nurse tends to report more missed care during shifts that are more demanding than their personal average. By contrast, a between‐nurse association would indicate that nurses who are habitually exposed to higher workloads report, on average, more missed care than nurses with lower habitual exposure (Connolly et al. 2025; Duman and Arikan 2026). These processes may have different explanations and implications. Within‐nurse fluctuations may reflect acute workload peaks, unexpected patient demands, disruptions, or temporary organizational pressures and could potentially be addressed through real‐time staffing adjustments and shift‐level support. Between‐nurse differences may instead reflect persistent inequalities in assignments, chronic understaffing, unit characteristics, role differences, or stable features of the work environment requiring broader organizational intervention (Al Muharraq et al. 2022; Griffiths et al. 2019).

A small but growing body of repeated‐measures and diary‐based research has begun to address this temporal dimension by examining how workload, task complexity, organizational accountability, and care processes vary across shifts (Suliman et al. 2026). However, the use of repeated observations or multilevel models does not by itself separate within‐nurse fluctuations from between‐nurse differences. Unless time‐varying workload variables are explicitly decomposed into person‐specific deviations and person‐level averages, the resulting coefficients may combine these two sources of variation, making interpretation difficult. Consequently, it remains unclear whether missed nursing care is primarily associated with sustained high workload, temporary workload peaks during specific shifts, or both (Suliman et al. 2026).

Missed nursing care is also unlikely to affect all nursing activities uniformly (Al Muharraq et al. 2022; Griffiths et al. 2019; Khajoei et al. 2025). When time and resources are constrained, nurses must continuously prioritize among simultaneous demands, and some activities may be more vulnerable to postponement or omission than others. Previous studies have shown that missed nursing care frequently involves fundamental, relational, educative, and planning activities, including mobilization, hygiene, oral care, communication with patients and families, emotional support, patient education, discharge preparation, and care planning (Ball et al. 2014, 2016). Nevertheless, surveillance and safety‐related activities may also be compromised when workload becomes excessive, particularly when nurses face interruptions, multiple unstable patients, or competing clinical priorities (Fan et al. 2026).

In this regard, different workload dimensions may plausibly affect different care domains (Fan et al. 2026). For example, a high number of assigned patients may reduce the time available for fundamental or relational care. In contrast, emotional and organizational pressures may be particularly relevant to communication, education, planning, and continuity activities. These propositions require empirical examination; however, differences between domains may also reflect measurement characteristics, the perceived urgency of specific tasks, or nurses' prioritization strategies rather than distinct workload mechanisms.

Despite the development of shift‐level and repeated‐measures research, three related questions therefore remain insufficiently resolved (Breno et al. 2025; Giannarou et al. 2026). First, patient‐count patient workload and perceived emotional‐organizational workload have rarely been examined simultaneously in a way that permits their independent contributions to be distinguished (Maghsoud et al. 2022). Second, repeated workload observations have seldom been explicitly decomposed into within‐nurse fluctuations and between‐nurse differences in average exposure (Ellehave et al. 2026). Third, limited evidence is available on whether these workload dimensions are associated with specific domains of missed nursing care rather than only with an overall score (Nazari et al. 2026). Addressing these gaps may provide a more precise account of when missed care occurs, which aspects of workload are most relevant, and which nursing activities are most vulnerable during demanding shifts. Accordingly, the primary aim of this study was to examine the mutually adjusted associations between patient count and perceived workload dimensions and the overall proportion of applicable nursing activities reported as missed in medical–surgical hospital units. Patient‐count workload indicators included the numbers of assigned, isolated, and patients requiring specialist care, whereas perceived workload dimensions included work rhythm and quantity, mental workload, emotional workload, and work organization. Analyses were based on repeated shift‐level observations clustered at the nurse level (within nurse). The secondary aims were to distinguish between within‐nurse associations (i.e., whether a nurse reported more missed care during shifts with higher‐than‐average workload) and between‐nurse associations reflecting differences in average workload across the sampled shifts, and to explore whether workload associations varied across missed‐care domains.

## 2 Methods

### 2.1 Design

This was an observational, repeated‐measures study based on shift‐level data collected from nurses working in medical‐surgical hospital units. The study is reported according to the Strengthening the Reporting of Observational Studies in Epidemiology (STROBE) recommendations for observational studies (von Elm et al. 2007). To enhance reporting transparency, the Supporting Information S1 provides detailed documentation of outcome coding and harmonization, domain construction, missingness and sample selection, hospital‐level distributions, model diagnostics, and complete estimates from the primary, sensitivity, within–between, item‐level, domain‐specific, and exploratory analyses (Tables S1–S11; Figures S1 and S2).

### 2.2 Setting and Participants

The study was conducted in 16 medical‐surgical units across six teaching hospitals located in northern, central, and southern Italy. Hospitals were recruited through convenience sampling among public and private institutions. Nurse executives at the participating hospitals were contacted and invited to take part in the study. Nurse executives who agreed to participate were asked to identify at least one medical and one surgical inpatient care unit providing care to patients admitted for general medical conditions or major surgical procedures. Wards were subsequently selected using a purposive sampling approach. Emergency, intensive or sub‐intensive care units, week or day‐care units, and units specifically designated for the care of patients with COVID‐19 were excluded.

Data were collected from nurses working in the selected medical and surgical inpatient care units. Nurses were eligible if they were registered nurses, worked full‐time in the selected unit, were directly involved in patient care, and had worked in the unit for at least 2 months. Newly employed nurses, nurses undergoing an orientation or training period, float nurses, and nurses working overtime or additional shifts were excluded. These criteria were established to minimize potential sources of variability in workload perception unrelated to usual unit conditions. Newly employed nurses and those still undergoing training may not yet be fully familiar with unit routines, organizational processes, team dynamics, and clinical procedures. Similarly, float nurses may have limited familiarity with unit‐specific workflows, communication practices, and the location or availability of materials and equipment, potentially increasing the time and effort required to perform routine activities. Nurses working overtime or additional shifts were excluded because extended working hours could independently influence perceived workload.

All registered nurses working in the selected units meeting the eligibility criteria were invited to participate in the study. Of the 269 nurses who met the eligibility criteria, 213 provided consent and participated in the study.

### 2.3 Sample Size

An a priori sample‐size calculation was conducted for the primary aim of examining the joint incremental contribution of patient‐count and perceived workload dimensions to overall missed nursing care. Because no routine closed‐form procedure was available for the planned clustered generalized estimating equation analysis, the initial calculation used a fixed‐model multiple‐regression approximation in G*Power 3.1, followed by adjustment for repeated observations within nurses.

The calculation assumed a small‐to‐moderate incremental effect of the seven workload indicators as a block (*f* ^2^ = 0.05), a two‐sided alpha level of 0.05, 80% power, seven tested workload predictors, and 16 total predictors, including nurse characteristics, shift, and hospital indicators. This yielded a requirement of 295 independent shift‐level observations. Assuming an average of 2.5 observations per nurse and a conservative within‐nurse correlation of 0.20, a design effect of 1.30 increased the requirement to 384 shift records. Allowing for 20% to account for incomplete observations, unequal cluster sizes, and non‐analyzable records yielded a target sample of 480 shift‐level observations, corresponding to approximately 192 nurses.

The final dataset comprised 502 shift‐level records from 213 nurses, while the primary complete‐case analysis included 480 records from 196 nurses, thereby meeting the planned analytical sample size. The calculation was intended to support the primary global workload analysis; within‐between decompositions and domain‐specific analyses were considered secondary and were not separately powered.

### 2.4 Data Collection

After the nurse executives identified the 16 participating units, the research team contacted the nurse managers. Meetings were held with nurse executives and nurse managers to present the study aims, procedures, and requirements and to obtain approval for data collection. Additional meetings were then conducted with unit nurses to explain the study and invite them to participate.

Registered nurses were instructed to complete the self‐administered survey on nursing workload and missed care immediately after the end of each randomly selected work shift. No additional post‐shift completion window was prespecified. The estimated 10–15 min referred to the time required to complete the questionnaire rather than to an allowable delay after shift completion. Data were collected during day or night shifts over three consecutive weeks at each participating unit between February 2021 and June 2023. Hospitals entered the study sequentially after the relevant local ethics and organizational approvals had been obtained. Data collection was subsequently conducted over three consecutive weeks in each participating unit; consequently, the hospital‐specific collection windows were staggered across the overall study period. Shifts were recorded using two prespecified categories, day and night, reflecting the 12‐h scheduling system represented in the participating settings. No separate evening category was collected, and evening and night shifts were therefore not combined post hoc for analysis. Each participant was assigned a unique numerical code to support data linkage and preserve confidentiality. Nurses received an individual link to the survey through their institutional email account. The questionnaire was administered via Google Forms and could be accessed on various devices and web browsers. Completion required approximately 10–15 min.

Participation was voluntary, and nurses could decide whether to complete the survey for each eligible observation. Data were collected and stored in accordance with procedures designed to protect confidentiality and data security.

### 2.5 Measurements

Data were collected using a structured questionnaire consisting of two sections.

The first section collected nurse‐, shift‐, and patient‐level characteristics. Nurse‐level variables included age, sex, and professional seniority. Nurses also reported the hospital, the unit they worked on (medical or surgical), and the shift (day or night). Shift‐level patient‐count workload indicators included the numbers of assigned, isolated, and patients requiring specialist care. Nurses rated the overall complexity of the patients under their care during the completed shift using a single ad hoc item developed for this study, with response options ranging from 0 (“not at all”) to 4 (“very much”). The item was not derived from an existing framework or validated scale. It represented a global nurse‐rated appraisal at the shift level and was not designed to distinguish medical complexity, patient acuity, dependency, or the complexity of nursing care generated by specific nursing diagnoses and activities.

The second section assessed perceived workload, work organization, and missed nursing care. Four scales from the questionnaire on the Experience and Evaluation of Work (QEEW 2.0) were used: the six‐item Pace and Amount of Work scale (reported here as work rhythm/quantity), the four‐item Mental Workload scale, the five‐item Emotional Workload scale, and the six‐item Work Organization scale (van Veldhoven et al. 2015). Items were rated from 0 (“never”) to 3 (“always”). Scale scores were calculated according to the QEEW 2.0 scoring instructions and linearly transformed to a 0–100 metric. Higher scores indicated greater work rhythm/quantity, mental workload, and emotional workload; for work organization, higher scores indicated poorer organization. The QEEW 2.0 documentation reports reliability coefficients (rho) of 0.86, 0.81, 0.80, and 0.76, respectively (van Veldhoven et al. 2015).

Missed nursing care was assessed using Section A of the Unfinished Nursing Care Survey (UNCS) developed by Bassi et al. (2020). The UNCS was developed and validated among nurses working in Italian acute‐care hospitals. In this study, the full 37‐activity version of Section A was used with permission from the authors. Nurses reported how frequently each activity had been left unfinished during the shift under assessment, rather than over the original seven‐working‐day reference period. This adaptation was introduced to align the outcome temporally with workload indicators referring to the same completed shift and to reduce recall and temporal aggregation across multiple working days. The 37 activities, response categories, and “not applicable” option were otherwise unchanged. However, the shift‐level adaptation has not undergone a separate psychometric validation and should not be assumed to be measurement‐equivalent to the original seven‐working‐day version. Responses ranged from 1 (“never”) to 5 (“always”). An additional response option, 6 (“not applicable in my setting”), was provided when an activity was not relevant to the clinical context (Bassi et al. 2020). Only Section A of the UNCS was administered.

#### 2.5.1 Outcome Variable

The primary outcome was the number of missed nursing care activities reported per shift, among applicable activities. During data preparation, the survey response 6 (“not applicable”) was recoded to 0. Values 1–5 were treated as applicable responses, values 3–5 were coded as missed, and 0 and missing or invalid responses were excluded from both numerator and denominator. Before outcome derivation, center‐stratified response distributions and item profiles were audited. The audit identified a consistent reversal of response direction across all 37 missed‐care items in Hospitals 4 and 5. Following this finding, members of the local research teams who had configured and administered the online questionnaires in these hospitals were contacted. They confirmed that when the online instrument was created, the five ordered response categories were programmed in the opposite numerical order to the original UNCS. The wording of the activity and the response categories had otherwise remained unchanged. Consequently, applicable values from 1 to 5 were recoded using 6 − x, corresponding to 1 → 5, 2 → 4, 3 → 3, 4 → 2, and 5 → 1. This deterministic recoding restored the original UNCS metric, ranging from 1 (“never”) to 5 (“always”); “not applicable” and missing responses were not altered. The correction was therefore based on confirmation of the online data‐capture coding by the researchers directly involved in questionnaire administration and was further supported by the distributional and item‐profile audit, rather than being selected solely on the basis of model results. All reported models use this harmonized missed‐care outcome (Table S1; Figure S1).

As secondary outcomes, missed nursing care was grouped into four clinically defined domains: fundamental mobility/self‐care, surveillance/safety/treatment, communication/education/relationship, and planning/coordination/continuity. For each domain, analyses used the number of missed activities over the number of applicable activities within that domain. Full item‐domain definitions are reported in Table S2. The primary missed‐care threshold treated values 3–5 as missed care; threshold sensitivity analyses also evaluated values 2–5 and 4–5 as alternative definitions (Table S1).

#### 2.5.2 Exposure Variables

The main exposure variables were patient‐count workload and perceived workload. Patient‐count workload indicators included the nurse‐reported numbers of assigned patients, isolated patients, and patients requiring specialist care. The ad hoc nurse‐rated patient‐complexity item was examined only as an additional shift‐level indicator in supplementary analyses and was not treated as an objective or validated measure of patient or nursing complexity. Perceived workload dimensions included work rhythm/quantity, mental workload, emotional workload, and work organization.

For repeated‐measures analyses, time‐varying workload variables were decomposed into within‐nurse and between‐nurse components. The within‐nurse component represented the deviation of a given shift from that nurse's own average workload. The between‐nurse component represented each nurse's average workload exposure across observed shifts. This decomposition follows the logic of within‐between or correlated random‐effects approaches for separating within‐person from between‐person associations in repeated‐measures data (Hamaker and Muthén 2020).

#### 2.5.3 Covariates

Models adjusted for nurse and shift characteristics considered clinically and analytically relevant: age, sex, professional seniority, shift, and hospital. Hospital was included as a fixed effect because only six hospitals were available and because the patient count was used to control for observed center differences rather than estimate a population distribution of hospital effects. Ward fixed effects were examined as a sensitivity analysis.

#### 2.5.4 Quantitative Variables

Continuous workload variables were standardized before modeling to facilitate comparison of effect sizes across predictors. The total missed‐care outcome was analyzed primarily as a grouped‐binomial outcome because the number of missed activities was constrained by the number of applicable activities.

### 2.6 Bias

Several potential sources of bias were considered. Missed nursing care and perceived workload were self‐reported, which may introduce recall, social‐desirability, response‐style, or common‐method bias.

To reduce recall bias, nurses were instructed to complete the questionnaire immediately after the selected work shift rather than retrospectively over longer periods. However, the elapsed time between shift completion and questionnaire submission was not separately recorded. Standardized written and verbal instructions were provided to all participants before data collection to ensure a consistent understanding of the study procedures and questionnaire completion.

To reduce social‐desirability bias, participation was voluntary, questionnaires were completed individually using a secure web‐based platform accessed through a personal survey link, and participants were identified only by a unique numerical code used for data linkage across repeated observations.

To minimize selection bias at the shift level, data collection was scheduled over three consecutive weeks in each participating unit, and nurses were invited to complete the questionnaire after randomly selected day or night shifts. However, participation in each eligible shift remained voluntary, and differential participation among nurses or shifts cannot be ruled out.

Nurses contributed different numbers of observations, creating within‐person correlation that required nurse‐clustered methods. This dependence was addressed analytically using GEE with nurse‐level clustering and robust standard errors. In addition, workload variables were decomposed into within‐nurse and between‐nurse components to distinguish changes occurring within individuals over time from differences between individuals.

### 2.7 Statistical Analysis

Data completeness, denominator structure, center‐specific response direction, and hospital‐level plausibility were assessed before analysis. Complete‐case exclusions were described by hospital and variable. Applicable activities and valid item responses were kept conceptually separate by auditing not‐applicable responses and missing responses independently. Missingness and complete‐case selection are summarized in Table S3, hospital‐level denominator patterns are reported in Table S4, and the direction‐harmonization audit is presented in Table S1 and Figure S1.

Descriptive statistics were used to summarize nurse‐level characteristics separately from shift‐level characteristics. Nurse‐level characteristics are reported for all unique nurses with available data, not weighted by the number of shifts contributed. Shift‐level variables included shift type, patient‐count workload indicators, perceived workload dimensions, applicable‐activity denominators, and missed‐care counts.

The primary model used a grouped‐binomial GEE with a logit link, nurse‐level clustering, empirical sandwich robust standard errors, an exchangeable working correlation, and hospital fixed effects, modeling missed versus non‐missed applicable activities. Analyses were conducted in R 4.6.1 using geepack 1.3.13. The primary model included 196 nurse clusters, 63 of which contributed one observation; the estimated working correlation was 0.185.

The main model estimated associations of patient‐count and perceived workload variables with the proportion of applicable activities missed, adjusting for age, sex, professional seniority, shift, and hospital fixed effects. A within‐between model then decomposed time‐varying workload variables into nurse‐specific means and shift‐specific deviations from each nurse‐specific mean, following a Mundlak/person‐mean approach. To address multiplicity in the primary model, the joint null hypothesis that all seven workload coefficients equaled zero was evaluated using a robust Wald test based on the empirical covariance matrix from the generalized estimating equation model. Secondary blockwise Wald tests evaluated the four perceived‐workload indicators and the three patient‐count indicators separately, with each block adjusted for the others and all model covariates. Benjamini–Hochberg *q*‐values were also calculated across the seven coefficient‐specific workload tests. Individual coefficients were interpreted with caution when they did not remain significant after adjustment for multiple testing. To assess the sensitivity of the within–between estimates to the sparse repeated‐measures structure, the model was refitted after restricting the sample to nurses who contributed at least two complete‐case shift observations.

Sensitivity and supplementary analyses included working‐independence GEE; exclusion of Hospitals 4 and 5; a model excluding age, sex, and professional seniority; item‐level GEE with missed‐care item fixed effects; negative binomial models with nurse‐clustered robust standard errors, including models with log(applicable activities) as an offset; domain‐specific grouped‐binomial GEE; alternative missed‐care thresholds; and exploratory categorical workload models. A ward‐fixed‐effects GEE was attempted but became non‐estimable due to quasi‐separation and was retained only for diagnostics. Benjamini‐Hochberg *q*‐values were calculated across 28 focal domain tests and across the four work rhythm/quantity domain estimates.

### 2.8 Ethical Considerations

The study was conducted in accordance with the Declaration of Helsinki. The study received approval from all the local Ethics Committees (Committee of University Campus Bio‐Medico of Rome on November 9, 2020 with the protocol number: Prot.: 95/20 OSS ComEt CBM; the Ethics Committee of ASL Lecce on May 14, 2021 with the protocol number: Verbale N. 62; the Ethics Committee “LAZIO 2” on June 9, 2021 with the protocol number N. 0116717/2021, the Ethics Committee “IRCCS ISMETT” on September 21, 2022 with the protocol number IRRB/13/22; the Ethics Committee “INSUBRIA” on October 11, 2022 with the verbal number N. 105). All participants received written information about the study and provided written informed consent. Those who declined consent were not enrolled. Anonymity was protected by assigning each participant a unique numerical code, and data access was restricted to the research team.

## 3 Results

### 3.1 Sample and Shift Characteristics

Of 269 eligible nurses, 213 (79.2%) consented and participated. The full dataset included 502 shift‐level records across six hospitals and 16 medical–surgical units; 501 records had a nurse identifier. The nurse‐level denominator was 213 nurses, while the primary complete‐case analytical sample included 480 observations from 196 nurses. Table 1 separates nurse‐level characteristics, reported for all unique nurses with available data, from shift‐level characteristics. Aggregate missingness and complete‐case selection are reported in Table S3.

**TABLE 1:** Nurse, shift, workload, and missed care characteristics.

Panel	Characteristic	Denominator	Value
Panel A. Nurse‐level characteristics	Nurses	213	213
	Nurses with more than one observation	213	136 (63.8)
	Observations per nurse, median (IQR)	213	2.0 (1.0–3.0)
	Age, mean (SD)	196	35.6 (10.1)
	Professional seniority in months, median (IQR)	199	61.0 (24.0–167.0)
	Female	196	169 (86.2)
	Male	196	27 (13.8)
Panel B. Shift‐level characteristics	Shift‐level observations	502	502
	Observations with nurse identifier	502	501 (99.8)
	Hospitals	502	6
	Units	502	16
	Day shift	502	291 (58.0)
	Night shift	502	211 (42.0)
	Number of assigned patients, median (IQR)	502	9.0 (7.0–10.0)
	Isolated patients, median (IQR)	502	0.0 (0.0–1.0)
	Specialist‐care patients, median (IQR)	502	2.0 (1.0–3.0)
	Patient complexity, median (IQR)	502	3.0 (2.0–3.0)
	Work rhythm/quantity, mean (SD)	501	43.0 (22.9)
	Mental workload, mean (SD)	501	78.4 (26.5)
	Mental workload, median (IQR)	501	91.7 (66.7–100.0)
	Emotional workload, mean (SD)	501	56.1 (16.7)
	Work organization, mean (SD)	501	47.6 (18.6)
	Missed care count, mean (SD)	502	6.5 (8.2)
	Missed care count, median (IQR)	502	3.0 (0.0–10.0)
	Zero missed care	502	150 (29.9)
	Applicable‐activity denominator, median (IQR)	502	37.0 (37.0–37.0)
	Observations with fewer than 37 applicable activities	502	87 (17.3)

In the primary complete‐case sample, 63 of 196 nurse clusters (32.1%) were singletons and therefore had no estimable within‐nurse deviation. Among the 133 complete‐case nurses with repeated observations, 123 showed within‐nurse variation in work rhythm/quantity. The nurse‐level intraclass correlation coefficient for work rhythm/quantity was 0.49 (Table S1).

### 3.2 Distribution of Missed Nursing Care

Across all observations, nurses reported a mean of 6.5 missed care activities per shift (SD = 8.2; median = 3; IQR = 0–10; range = 0–37). Overall, 150 observations (29.9%) had no missed care. The number of applicable activities was unchanged by direction harmonization: 87 observations (17.3%) had fewer than 37 applicable activities, with a minimum denominator of 16. The denominator audit identified 253 not‐applicable responses and 10 missing‐item responses across 18,574 item slots (Table S4).

Mean missed‐care counts ranged from 4.5 (Hospital 4) to 10.8 (Hospital 2), and the proportion of shifts with no missed care ranged from 13.3% (Hospital 2) to 44.7% (Hospital 4); Hospitals 4 and 5 fell within the range of the other centers on both measures (Table S4). The L1 distance from the pooled reference distribution was 0.26 in Hospital 4 and 0.11 in Hospital 5; the item‐profile Spearman correlations were 0.61 and 0.68, respectively. Mental workload remained substantially lower in Hospitals 4 and 5 (Tables S1 and S4; Figure S1).

### 3.3 Primary Grouped‐Binomial GEE Model

The complete‐case sample for the primary model included 480 observations from 196 nurses (see Table 2). The seven workload indicators were jointly associated with missed nursing care (robust Wald *χ* ^2^[7] = 22.95, *p* = 0.002). In secondary blockwise analyses, the four perceived‐workload indicators were jointly associated with missed care after adjustment for the patient‐count indicators and remaining covariates (*χ* ^2^[4] = 16.90, *p* = 0.002), whereas the three patient‐count indicators were not clearly associated after adjustment for perceived workload (*χ* ^2^[3] = 4.09, *p* = 0.252). At the coefficient level, work rhythm/quantity was the only workload indicator with a nominal *p*‐value below 0.05 (OR 1.24 per SD, 95% CI 1.03–1.49; *p* = 0.023); however, it did not remain statistically significant after Benjamini–Hochberg correction across the seven workload coefficients (*q* = 0.159). No individual workload coefficient remained significant at *q* < 0.05. Complete primary‐model estimates and model diagnostics are provided in Tables S5 and S6.

**TABLE 2:** Primary grouped‐binomial GEE model for total missed nursing care.

Predictor	OR (95% CI)	*p*
Work rhythm/quantity	1.24 (1.03–1.49)	0.023
Mental workload	0.94 (0.78–1.12)	0.475
Emotional workload	1.12 (0.95–1.32)	0.174
Work organization	1.00 (0.83–1.19)	0.964
Assigned patients	1.14 (0.94–1.40)	0.192
Isolated patients	0.92 (0.82–1.04)	0.166
Specialist‐care patients	1.04 (0.88–1.22)	0.637

*Note:* The model used harmonized missed versus non‐missed applicable activities, nurse clustering, empirical sandwich‐robust standard errors, an exchangeable working correlation, and hospital fixed effects. Continuous predictors are standardized. Model *n* = 480 shift records from 196 nurse clusters; working correlation = 0.185. Individual coefficient *p* values are nominal. The robust omnibus Wald test of the seven workload coefficients yielded *χ* ^2^(7) = 22.95, *p* = 0.002. Benjamini–Hochberg *q* values were calculated across the seven workload coefficients; none remained significant at *q* < 0.05, and the *q* value for work rhythm/quantity was 0.159.

Abbreviations: CI = confidence interval, GEE = generalized estimating equations, OR = odds ratio.

Following harmonization, none of the hospital fixed‐effect estimates was extreme. Compared with Hospital 1, the ORs were 1.83 (95% CI 0.96–3.46; *p* = 0.065) for Hospital 2, 1.12 (95% CI 0.65–1.93; *p* = 0.670) for Hospital 3, 0.98 (95% CI 0.32–2.99; *p* = 0.978) for Hospital 4, 1.44 (95% CI 0.59–3.49; *p* = 0.421) for Hospital 5, and 1.27 (95% CI 0.80–2.02; *p* = 0.318) for Hospital 6. The harmonization decision was based on confirmation from the local research teams responsible for configuring and administering the online questionnaires; the absence of extreme post‐harmonization hospital estimates is reported as an additional consistency check and was not used to determine the recoding.

### 3.4 Sensitivity Analyses

The nominal work rhythm/quantity coefficient remained directionally consistent and had *p* values below 0.05 in several shift‐level sensitivity specifications: excluding Hospitals 4 and 5 (OR 1.26, 95% CI 1.03–1.55; *p* = 0.022), using a working‐independence correlation structure (OR 1.29, 95% CI 1.07–1.56; *p* = 0.009), and omitting age, sex, and professional seniority so that 499 records could be retained (OR 1.24, 95% CI 1.03–1.48; *p* = 0.021). A negative binomial model with an offset for the number of applicable activities produced a similar estimate (IRR 1.21, 95% CI 1.03–1.41; *p* = 0.021). Complete estimates for these shift‐level sensitivity analyses are reported in Table S6 and summarized in Figure S2. These sensitivity analyses indicate specification consistency but do not alter the exploratory status of the individual coefficient after correction across the seven primary workload tests.

Not all specifications reproduced this coefficient‐level pattern. At the item level, the working‐independence model produced a nominal association (OR 1.31, 95% CI 1.07–1.60; *p* = 0.009), whereas the exchangeable model was imprecise (OR 1.06, 95% CI 0.83–1.35; *p* = 0.629), indicating sensitivity to the assumed correlation structure. Complete item‐level estimates are reported in Table S8. The ward fixed‐effects GEE became non‐estimable due to quasi‐separation and is therefore reported as a diagnostic rather than a robustness test; the related diagnostic information is provided in Table S5.

Threshold analyses were directionally consistent at the liberal cut point but diverged at the strict one. With values 2–5 coded as missed, work rhythm/quantity remained associated (OR 1.22, 95% CI 1.05–1.43; *p* = 0.011), whereas patient count was not (OR 1.16, 95% CI 0.98–1.38; *p* = 0.092). With values 4–5 coded as missed, the six‐hospital pattern reversed: work rhythm/quantity became imprecise (OR 1.27, 95% CI 0.99–1.64; *p* = 0.061) while assigned patient count became associated (OR 1.31, 95% CI 1.01–1.70; *p* = 0.040). This reversal was not reproduced when Hospitals 4 and 5 were excluded, in which work rhythm/quantity remained associated (OR 1.40, 95% CI 1.06–1.83; *p* = 0.016), whereas patient count was not (OR 1.28, 95% CI 0.96–1.71; *p* = 0.089). The strict threshold substantially reduced the number of events per shift, resulting in less precise estimates. These findings are therefore reported as sensitivity to outcome definition rather than as evidence of substantive differences between workload indicators (Table S1).

### 3.5 Within‐Between Workload Associations

**FIGURE 1:**
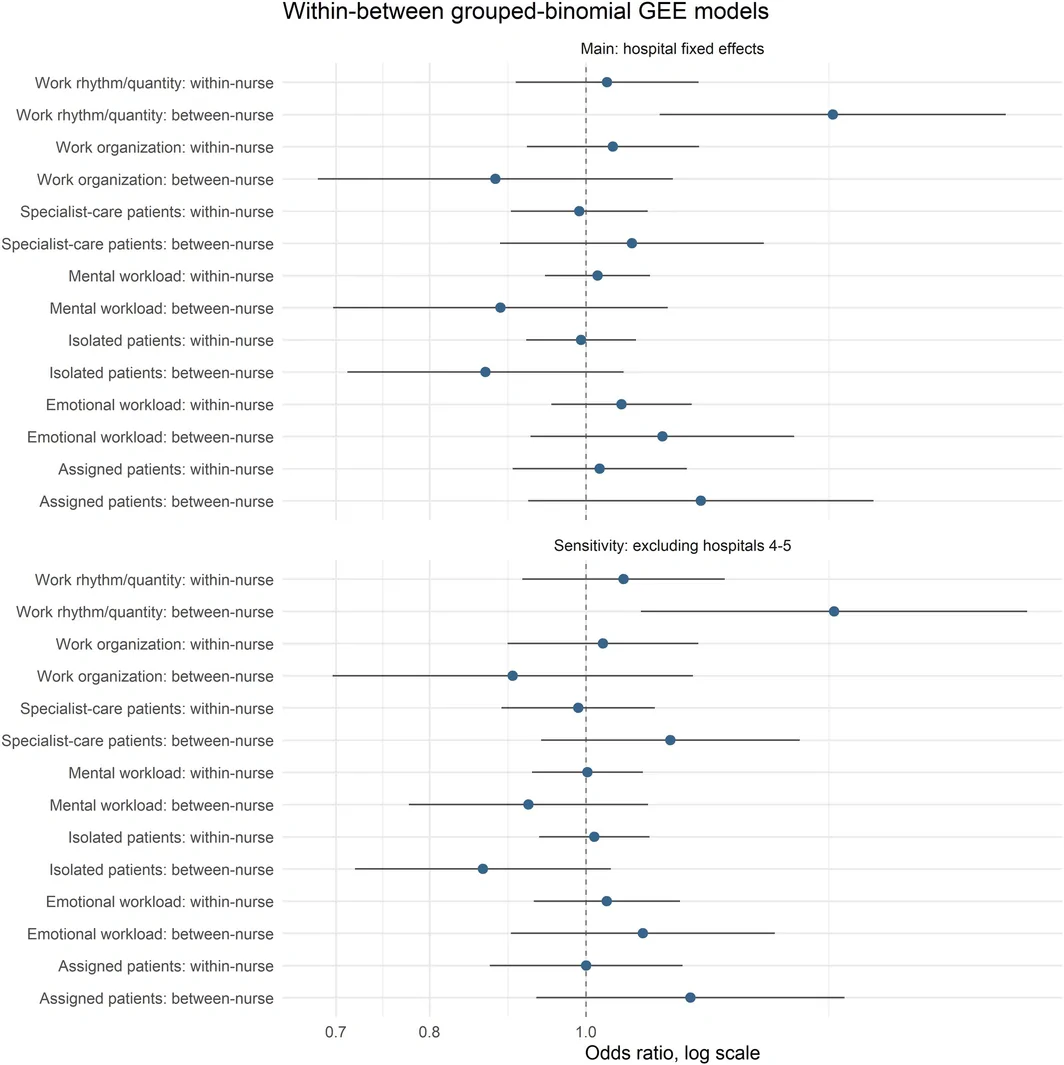
Within‐between grouped‐binomial GEE model estimates. Points represent odds ratios and 95% confidence intervals from the harmonized grouped‐binomial GEE analyses.

In the within‐between grouped‐binomial GEE model, the clearest signal concerned between‐nurse differences (see Table 3; Figure 1). Nurses with a higher average work rhythm/quantity across sampled shifts reported more missed care (OR 1.42, 95% CI 1.11–1.82; *p* = 0.005), whereas shift‐specific deviations from a nurse's own average were not clearly associated (OR 1.03, 95% CI 0.90–1.17; *p* = 0.649). No other workload dimension showed a clear association in either component (see Figure 1). Full estimates from the primary and repeated‐observation‐restricted within–between models are provided in Table S7.

**TABLE 3:** Within‐between grouped‐binomial GEE estimates for total missed nursing care.

Predictor	Within‐nurse OR (95% CI)	*p*	Between‐nurse OR (95% CI)	*p*
Work rhythm/quantity	1.03 (0.90–1.17)	0.649	1.42 (1.11–1.82)	0.005
Mental workload	1.02 (0.94–1.10)	0.664	0.89 (0.70–1.12)	0.317
Emotional workload	1.05 (0.95–1.16)	0.320	1.12 (0.92–1.35)	0.255
Work organization	1.04 (0.92–1.18)	0.538	0.88 (0.68–1.13)	0.318
Assigned patients	1.02 (0.90–1.15)	0.756	1.18 (0.92–1.51)	0.192
Isolated patients	0.99 (0.92–1.07)	0.864	0.87 (0.71–1.06)	0.154
Specialist‐care patients	0.99 (0.90–1.09)	0.852	1.07 (0.88–1.29)	0.494

*Note:* Within‐nurse estimates represent shift‐specific deviations from a nurse‐specific mean; between‐nurse estimates represent differences in nurses' average workload across sampled shifts. Model *n* = 480 shift records from 196 nurse clusters.

Abbreviations: CI = confidence interval, GEE = generalized estimating equations, OR = odds ratio.

In the sensitivity analysis restricted to nurses with at least two complete‐case observations, 417 shifts from 133 nurses were retained. The within‐nurse work rhythm/quantity estimate was essentially unchanged (OR 1.03, 95% CI 0.90–1.18; *p* = 0.646). The between‐nurse estimate remained positive but was attenuated and became imprecise (OR 1.32, 95% CI 1.00–1.75; *p* = 0.054), compared with OR 1.42 (95% CI 1.11–1.82; *p* = 0.005) in the full complete‐case sample. Thus, the directional within–between contrast was retained, but the evidence for the between‐nurse coefficient weakened after singleton nurses were excluded. A nominal between‐nurse coefficient was also observed for assigned patient count (OR 1.46, 95% CI 1.05–2.01; *p* = 0.023), but it did not remain significant after Benjamini–Hochberg correction across the 14 within–between workload coefficients (*q* = 0.320). No coefficient in the restricted model remained significant at *q* < 0.05.

### 3.6 Domain‐Specific Missed Care

Domain‐specific analyses showed that missed care was not evenly distributed across activities. The highest mean rate was observed for communication, education, and relationship‐based care (0.234), followed by fundamental mobility/self‐care (0.218), planning, coordination, and continuity (0.161), and surveillance, safety, and treatment (0.132). Domain definitions and item allocations are reported in Table S2.

Complete domain‐specific model estimates, including all seven focal predictors and multiplicity‐adjusted *q*‐values, are reported in Table S9.

**FIGURE 2:**
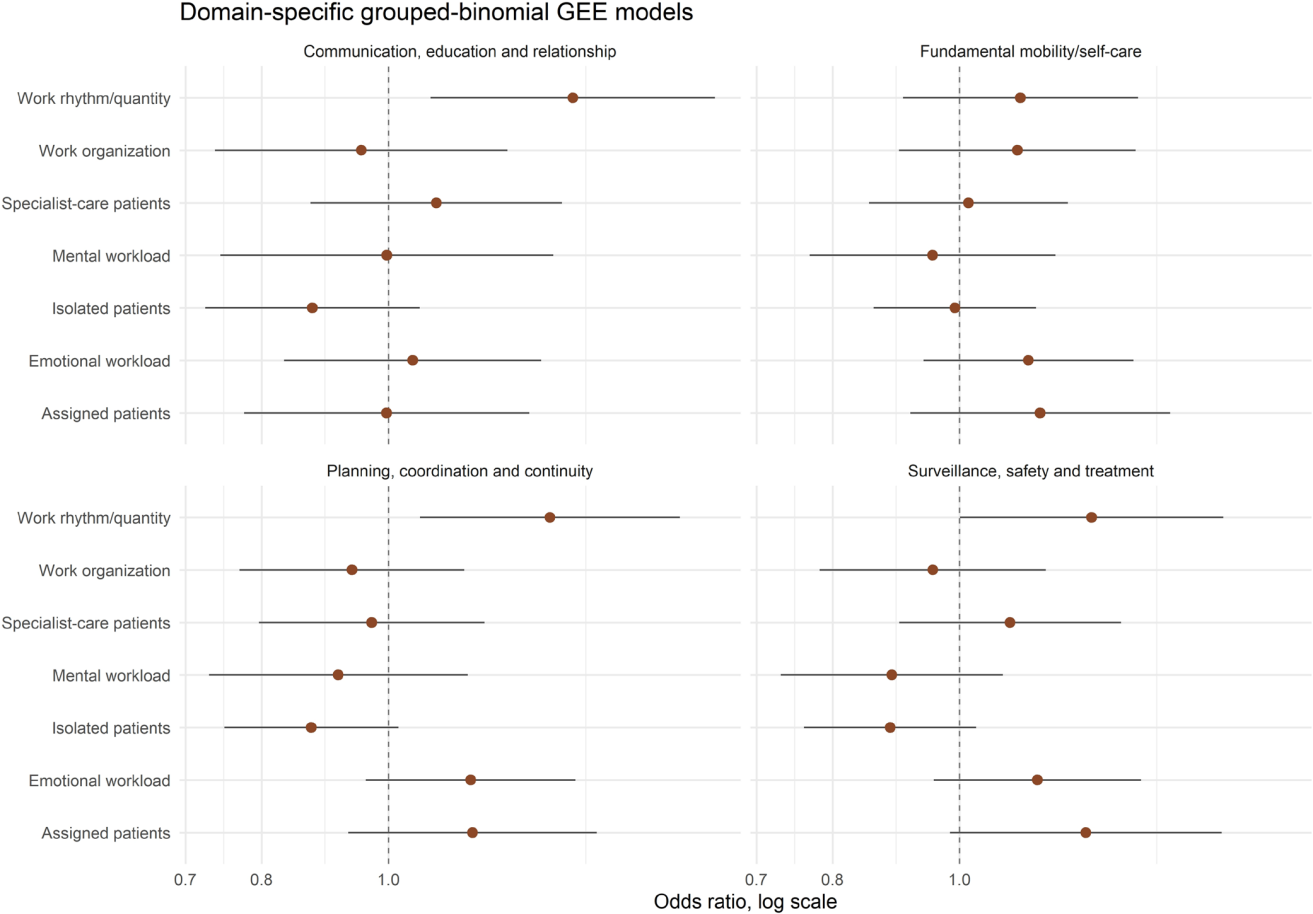
Domain‐specific grouped‐binomial GEE model estimates. Points represent odds ratios (ORs), and horizontal lines represent 95% confidence intervals, estimated using domain‐specific grouped‐binomial GEE models with nurse clustering and hospital fixed effects. The communication, education, and relationship model included 479 observations, whereas all other domain‐specific models included 480 observations.

Figure 2 summarizes the domain‐specific grouped‐binomial GEE estimates. These analyses are exploratory and should be interpreted alongside the full *q*‐value table in the supplement.

Work rhythm/quantity was associated with missed communication/education/relationship care (OR 1.38, 95% CI 1.08–1.78; *p* = 0.011), planning/coordination/continuity (OR 1.33, 95% CI 1.06–1.67; *p* = 0.015), and, more weakly, surveillance/safety/treatment (OR 1.26, 95% CI 1.00–1.59; *p* = 0.049). None remained below *q* = 0.05 across all 28 focal tests. In the focused four‐domain work rhythm/quantity family, communication and planning remained below *q* = 0.05, whereas surveillance had *q* = 0.065. Fundamental mobility/self‐care was not clearly associated with work rhythm/quantity.

### 3.7 Supplementary Patient‐Count and Nurse‐Rated Complexity Analyses

Supplementary analyses categorized the three patient‐count workload indicators and the ad hoc nurse‐rated patient‐complexity item into exploratory classes: assigned patients, isolated patients, specialist‐care patients, and nurse‐rated complexity. These analyses should be interpreted cautiously because category distributions differed strongly by hospital and common support was limited. Cell counts by hospital and category are provided in Table S10.

The ad hoc nurse‐rated patient‐complexity item was associated with all four perceived workload dimensions in supplementary linear models adjusted for patient‐count workload indicators, nurse characteristics, shift, and hospital: work rhythm/quantity (standardized beta 0.45, 95% CI 0.33–0.56; *p* < 0.001), mental workload (beta 0.13, 95% CI 0.06–0.21; *p* < 0.001), emotional workload (beta 0.28, 95% CI 0.16–0.41; *p* < 0.001), and work organization (beta 0.35, 95% CI 0.26–0.44; *p* < 0.001). However, these categorical workload thresholds remain exploratory rather than clinically established. Complexity‐workload models are reported in Table S11.

## 4 Discussion

The primary inferential finding of this multicenter repeated‐measures study was that the seven workload indicators were jointly related to missed nursing care. Secondary blockwise analyses indicated that this global signal was evident in the perceived‐workload block but not in the patient‐count block after mutual adjustment. Work rhythm/quantity showed the clearest individual coefficient and was directionally consistent across most shift‐level sensitivity analyses; however, it did not remain statistically significant after correction across the seven coefficient‐specific tests. Similarly, none of the domain‐specific associations remained significant when all 28 focal tests were considered together. The findings therefore support the relevance of perceived workload as a multidimensional construct but do not establish that work rhythm/quantity independently contributes information beyond patient count. Its coefficient should instead be interpreted as a focused hypothesis for future confirmatory research.

Previous studies have consistently linked missed nursing care with staffing adequacy, nursing resources, work environment, and workload (Ausserhofer et al. 2014; Ball et al. 2014, 2016; Griffiths et al. 2018; Recio‐Saucedo et al. 2018; Sarpong et al. 2026). Our results are broadly consistent with this literature and extend it by jointly examining patient count and perceived workload indicators within a repeated shift‐level framework. Although work rhythm/quantity produced the clearest individual coefficient, the multiplicity‐adjusted evidence supports the perceived‐workload block collectively rather than a specific comparative advantage of work rhythm/quantity over patient count. This pattern converges with the strongest available shift‐level evidence: in a single‐center neonatal study, subjective workload measured with the NASA Task Load Index was associated with missed care in all fitted models, whereas staffing‐ratio effects were less consistent once workload measures were modeled jointly (Tubbs‐Cooley et al. 2019); the same research group subsequently replicated this finding across ten neonatal units and 11,364 matched nurse–infant shifts, in which higher subjective workload remained associated with missed care for all 17 types of care in joint models while the effects of staffing ratios diminished (Tubbs‐Cooley et al. 2025). Comparable divergence between assessment approaches has been reported in adult intensive care, where three different workload assessment methods yielded different estimates of missed‐care prevalence and were not interchangeable (Caillet et al. 2025), and in nested diary designs in which subjective workload moderated rather than merely paralleled the effect of patient‐count workload on nurse‐reported missed care (Bayadsi et al. 2025). Our findings extend this evidence to adult medical–surgical units, in which the balance between patient‐count and perceived workload indicators had not previously been examined with an explicit within–between decomposition.

This does not mean that patient‐count workload or patient mix is unimportant. In supplementary analyses, the single ad hoc nurse‐rated patient‐complexity item was associated with all four perceived workload dimensions. Because complexity and perceived workload were reported concurrently by the same nurse, this pattern may reflect a meaningful global appraisal of shift demands, but it may also be partly influenced by common‐method variance and does not establish the construct validity of the complexity item. In particular, this global rating cannot distinguish general or medical patient complexity from nursing complexity, which may vary among medically or administratively similar patients according to nursing diagnoses, nursing actions, care intensity, and resource requirements (Cesare et al. 2024, 2026; Sanson et al. 2019). Nurse–patient ratios remain robustly associated with mortality, adverse events, and care quality at the system level (Aiken et al. 2014; Fernández‐Sánchez et al. 2025; Griffiths et al. 2019), and there is active methodological work on how ratios and capacity should be operationalized in practice (Havaei et al. 2026; Ware et al. 2024). A plausible hypothesis is therefore that patient mix and nursing care requirements contribute to perceived demand through mechanisms not captured by simple patient counts. However, the categorical thresholds did not show simple monotonic patterns and had limited common support across hospitals, so they should remain supplementary and exploratory unless externally justified by clinical or regulatory criteria. Because mediation was not formally tested, this upstream interpretation should be regarded as a conceptual hypothesis rather than an estimated pathway.

Mental workload requires separate caution. It remained very high overall (median 91.7; upper quartile 100). The workload blocks were not directionally reversed: reversing them worsened distributional agreement, and mental workload remained positively correlated with work rhythm/quantity within every hospital. The residual center difference may therefore reflect a different version, anchor, administration procedure, or genuine context for the mental‐workload block. Mental workload was not independently associated with missed care in the adjusted model and should not be interpreted as protective.

### 4.1 Novelty of the Within–Between Decomposition

The within–between decomposition remains an important methodological feature, but the interpretation should be modest. Repeated observations alone do not separate person‐level from occasion‐level effects: unless time‐varying exposures are explicitly decomposed into person means and person‐mean‐centered deviations, regression coefficients conflate the two sources of variation and are not interpretable as either (Curran and Bauer 2011; Hamaker and Muthén 2020; Neuhaus and Kalbfleisch 1998). To our knowledge, this decomposition has rarely been applied to shift‐level missed‐care data in adult medical–surgical units, even in the small number of prospective repeated‐measures studies available (Bayadsi et al. 2025; Suliman et al. 2026; Tubbs‐Cooley et al. 2025).

In the exploratory within–between analysis, the largest individual estimate concerned the between‐nurse work rhythm/quantity component: nurses with higher average reported work rhythm/quantity across the sampled shifts also reported more missed care. This should not be described as sustained or chronic exposure because the median number of observations per nurse was only two, and person means may partly reflect the particular shifts sampled, stable unit assignment, role, response style, or unmeasured organizational factors. With few observations per person, the person mean is itself estimated with substantial sampling error, and the between‐nurse coefficient may absorb stable person‐level and contextual confounding that the within‐nurse coefficient excludes by construction (Curran and Bauer 2011).

The within‐between pattern retains a competing response‐style interpretation. Because perceived work rhythm/quantity and missed care were reported by the same nurse at the same time, stable reporting tendencies could inflate the between‐nurse association while within‐nurse centering removes part of that tendency. The contrast between the between‐nurse estimate (OR 1.42) and the within‐nurse estimate (OR 1.03) is compatible with stable exposure or context differences, but also with response‐style or common‐method bias. The work rhythm/quantity ICC of 0.49 and 63 singleton nurse clusters further limit interpretation (Table S1). Restricting the analysis to nurses with repeated observations preserved the direction of the within–between contrast but attenuated the between‐nurse work rhythm/quantity estimate, whose confidence interval included the null. This finding indicates that singleton nurses contributed information to the original between‐nurse estimate and further limits its interpretation as evidence of habitual workload exposure. The restricted analysis therefore supports a hypothesis‐generating interpretation rather than a stable between‐nurse effect.

This pattern has practical implications, but they are primarily hypothesis‐generating. Between‐nurse differences may reflect recurring assignment patterns, unit routines, staffing models, or individual reporting tendencies, all of which have been described as determinants of unfinished care in qualitative and review evidence (Ellehave et al. 2026; Fan et al. 2026; Gong et al. 2025). Future studies with more repeated observations per nurse are needed before distinguishing persistent exposure from sampling variability or stable contextual differences.

At the same time, the absence of clear within‐nurse associations should be interpreted cautiously and not as evidence of absence. Although 133 nurses in the complete‐case sample contributed repeated observations, the number with actual within‐nurse variation differed across predictors: for example, 123 varied in work rhythm/quantity, 92 in mental workload, 67 in emotional workload, and 67 in isolated patients. Limited within‐person variation reduces the effective information available for the within‐nurse contrast and may have attenuated these estimates toward the null (Hamaker and Muthén 2020).

### 4.2 Domain‐Specific Patterns Reveal Different Forms of Vulnerability

A further contribution of the study is the examination of missed nursing care by clinically interpretable domains rather than exclusively as a total count. Composite missed‐care scores implicitly assume that omissions are exchangeable across activities, whereas a recent systematic review found that distinct missed‐care activities are associated with distinct patient outcomes: omissions in monitoring, basic care, and treatment were linked to falls, readmissions, infections, and mortality, whereas omissions in communication and fundamental care were more strongly linked to reduced patient satisfaction (He et al. 2025). Domain‐level analysis is therefore not merely a descriptive refinement but a condition for outcome‐relevant interpretation.

One additional pattern deserves attention: fundamental mobility/self‐care had the second‐highest descriptive missed‐care rate, but it was the only domain without a clear association with work rhythm/quantity. This may indicate that omissions in this domain are driven by mechanisms other than shift pace, such as prioritization, dependency, staffing mix, local routines, or documentation practices.

However, the domain‐specific findings require caution. They involve multiple related tests, and no association remained below *q* = 0.05 when all 28 focal‐domain tests were adjusted together, although a secondary, focused work rhythm/quantity analysis across the four domains was more supportive. These results should therefore be framed as patterned exploratory evidence rather than definitive domain‐specific effects.

These findings support a differentiated interpretation of missed care, but they do not yet establish operational thresholds. Some omissions may reflect the global tempo and quantity of work, whereas others may depend on patient mix, interruptions, unit routines, professional values, or local documentation practices (Connolly et al. 2025; Ellehave et al. 2026; Nazari et al. 2026; Nobahar et al. 2023). Future research should test focused, theoretically prioritized clinical thresholds with adequate hospital‐level common support.

### 4.3 Implications for Nursing Management

The results question the adequacy of workload surveillance based exclusively on patient counts or formal staffing ratios. These indicators remain necessary and are associated with patient outcomes at the system level (Aiken et al. 2014; Griffiths et al. 2019), but future workload surveillance research should examine them alongside multidimensional perceived workload and more granular indicators of patient acuity, dependency, isolation burden, specialist‐care needs, nursing care requirements, and unit context. The same conclusion has been drawn from large shift‐level neonatal data, where high subjective workload was identified as a target for workload reduction alongside staffing ratios rather than as a substitute for them (Tubbs‐Cooley et al. 2025). Monitoring missed care itself is defensible as a routine quality and safety indicator, given its documented associations with patient satisfaction, infections, falls, and readmissions (He et al. 2025; Recio‐Saucedo et al. 2018), and its proposed role as a proximal indicator of staffing adequacy (Griffiths et al. 2018). Before translating the findings into operational thresholds, however, hospitals should verify that workload and missed‐care instruments are scored and interpreted equivalently across centers (Palese et al. 2021).

The domain‐specific findings suggest that prevention strategies may need to protect communication, education, planning, and surveillance activities during high‐paced shifts, since these domains carry distinct outcome implications (He et al. 2025). Where feasible, nurse‐reported measures should be complemented by objectively recorded process indicators, such as the timeliness of routinely documented vital‐signs observations, which have been shown to be sensitive to staffing levels and do not share the response‐style limitations of self‐report (Redfern et al. 2019). The categorical patient‐count workload findings are better treated as prompts for local audit than as ready clinical thresholds, because support across hospitals was uneven and raw category–outcome patterns were confounded by hospital.

### 4.4 Strengths and Limitations

This study has several strengths. It used repeated shift‐level observations, examined patient‐count and perceived workload jointly, decomposed within‐ and between‐nurse exposure components, modeled missed versus non‐missed applicable activities, and investigated an implausible center pattern using convergent distributional and item‐profile checks, followed by direct confirmation from the local research teams responsible for configuring and administering the online questionnaires, before harmonization. Results were then reassessed using alternative correlation structures, exclusion of Hospitals 4 and 5, a broader sample without demographic covariates, item‐level models, count models, and alternative missed‐care thresholds. Reporting followed STROBE recommendations (von Elm et al. 2007).

Several limitations must be acknowledged. First, the observational design precludes causal inference. Second, missed care and perceived workload were concurrent self‐reports and remain vulnerable to common‐method and response‐style bias (Podsakoff et al. 2003). In addition, shortening the UNCS reference period from seven working days to one completed shift improved temporal correspondence with the shift‐level exposures but constituted an adaptation of the validated instrument. Its measurement properties were not reassessed in the present sample; therefore, equivalence with the original UNCS cannot be assumed, and comparisons with studies using the original reference period should be made cautiously. Although the reversed online coding in Hospitals 4 and 5 was confirmed by the local research teams and the deterministic recoding restored the original UNCS response direction, the need for post‐collection harmonization remains a data‐quality limitation. Third, the mental‐workload block remained atypically low in Hospitals 4 and 5 and may not be measurement‐equivalent. In the overall sample, the mental‐workload distribution also showed a potential ceiling effect, with a median of 91.7 and an upper quartile of 100. This restricted upper‐range variability may have reduced the measure's ability to discriminate among nurses reporting high mental workload and attenuated its estimated association with missed care; the null coefficient should therefore be interpreted cautiously rather than as evidence of no relationship. Fourth, patient complexity was represented by a single ad hoc global nurse rating without separate psychometric validation. The item did not distinguish general or medical patient complexity from nursing complexity and may share response‐method variance with the perceived‐workload measures collected concurrently from the same respondent. Fifth, 22 observations were excluded from the complete‐case model, although their corrected missed‐care mean was similar to that of included observations (6.0 vs. 6.5), and the no‐demographics sensitivity analysis retained 499 records and produced a similar work rhythm/quantity estimate. Sixth, the item‐level exchangeable GEE was imprecise and the ward fixed‐effects GEE was non‐estimable because of quasi‐separation, showing that results are not invariant to every specification. Seventh, the omnibus test addressed multiplicity for the global workload hypothesis but did not identify which specific workload dimension accounted for the association. No individual workload coefficient remained statistically significant after Benjamini–Hochberg correction; coefficient‐specific, blockwise, within–between, and domain findings should therefore be interpreted as exploratory and require replication. Finally, repeated observations were sparse, one record lacked a nurse identifier, shift was recorded only as a binary day/night variable without exact start and end times, and generalizability beyond six Italian hospitals is uncertain.

### 4.5 Future Research

Future studies should replicate this approach using a larger number of hospitals and more repeated observations per nurse, so that person means approximate habitual exposure rather than the particular shifts sampled (Curran and Bauer 2011; Hamaker and Muthén 2020). Intensive shift‐level designs with matched nurse–patient records provide a feasible template (Tubbs‐Cooley et al. 2025), as do prospective repeated‐measures field studies incorporating task complexity and accountability (Suliman et al. 2026). Richer shift‐level workload measurement should include patient acuity and dependency, admissions and discharges, interruptions, documentation burden, isolation precautions, and specialist‐care requirements. Future studies should distinguish general patient or medical complexity from nursing complexity and incorporate standardized nursing data to capture nursing diagnoses, nursing care needs, nursing activities, care intensity, and resource requirements that may explain workload differences not adequately reflected by patient counts or broad global ratings of patient complexity (Cesare et al. 2024, 2026; Sanson et al. 2019). Wherever possible, nurse‐reported outcomes should be triangulated with objectively recorded care‐process indicators derived from routine electronic data (Redfern et al. 2019). Future analyses should define domain‐testing families a priori, formally verify measurement equivalence across hospitals before pooling (Palese et al. 2021), and consider item‐level or beta‐binomial mixed models when item‐level data and sample size permit.

## 5 Conclusion

The seven workload indicators were jointly associated with missed nursing care, and secondary blockwise analyses indicated that the global signal was evident in the perceived‐workload block rather than the patient‐count block. Work rhythm/quantity produced the clearest individual coefficient, but it did not remain statistically significant after correction across the seven workload indicators. Its between‐nurse component and the domain‐specific findings should also be considered exploratory. These results support multidimensional, repeated shift‐level workload assessment as a direction for future research but do not establish work rhythm/quantity as an independent or operational workload marker. The observational design, concurrent self‐report measures, and sparse repeated observations preclude causal interpretation.

## Funding

The authors have nothing to report.

## Disclosure

*Clinical Resources*: World Health Organization. *Global Patient Safety Action Plan 2021–2030* (https://www.who.int/publications/i/item/9789240032705). Agency for Healthcare Research and Quality, Patient Safety Network. *Nursing and Patient Safety* (https://psnet.ahrq.gov/primer/nursing‐and‐patient‐safety).

## Conflicts of Interest

The authors declare no conflicts of interest.

## Supporting information


**Table S1:** Exposure clustering, missed‐care threshold sensitivity, and CureM direction‐harmonization audit.
**Table S2:** Missed‐care domain definitions and item allocation.
**Table S3:** Missingness and complete‐case selection.
**Table S4:** Hospital‐level distributions after missed care direction harmonization and denominator audit.
**Figure S1:** Pooled CureM response distributions before and after direction harmonization. The original Hospital 4–5 profiles were reflected relative to Hospitals 1, 2, 3, and 6; recoding only CureM values 1–5 as 6‐x aligned their distributions with the reference profile. Workload items were not recoded.
**Table S5:** GEE model diagnostics.
**Table S6:** Primary grouped‐binomial GEE and shift‐level sensitivity analyses after CureM direction harmonization.
**Figure S2:** Work rhythm/quantity odds ratios across estimable shift‐level grouped‐binomial GEE sensitivity models after CureM direction harmonization. The ward fixed‐effects GEE is omitted because quasi‐separation made its coefficients non‐estimable.
**Table S7:** Within‐between grouped‐binomial GEE model.
**Table S8:** Item‐level GEE sensitivity models.
**Table S9:** Domain‐specific grouped‐binomial GEE models.
**Table S10:** Common support for exploratory objective workload categories.
**Table S11:** Patient complexity and perceived workload dimensions.

## Data Availability

The de‐identified data and R code supporting the findings of this study are available from the corresponding author upon reasonable request, subject to approval by the relevant institutions and applicable ethical and data‐protection requirements. The data are not publicly available because they include detailed nurse‐ and hospital‐level information subject to institutional data‐sharing restrictions.
