# Supplementary material for: Patient‐Count and Perceived Workload Associations With Missed Nursing Care: A Multicenter Repeated‐Measures Study
Source: J Nurs Scholarsh. 2026 Sep 8;58(5):e70135. doi: 10.1111/jnu.70135 (PMC13551504; doi:10.1111/jnu.70135)
Supplement: Supplementary file 1 [file JNU-58-0-s001.docx]

**Supplementary Materials**

Missed nursing care, workload, and six-hospital repeated shift-level dataset

# Supplementary Methods and Notes

**Dataset:** Six-hospital dataset: 502 shift-level records from 213 nurses; primary complete-case model: 480 records from 196 nurses.

**Primary model:** Grouped-binomial GEE for missed versus non-missed applicable activities, clustered by nurse, with hospital fixed effects.

**Privacy:** This editorial supplement excludes row-level excluded-record details and nurse-level inconsistency sheets to avoid unnecessary disclosure of pseudonymised identifiers.

**Data harmonization**: The 37 missed care items (CureM items) in Hospitals 4 and 5 were recoded from x to 6-x for values 1-5 after convergent distributional and item-profile evidence of reversed direction, confirmed by authors who collected data in those centres. Value 0, missing responses, and all workload items were unchanged.

Note: All tables and figures in this supplement use the harmonized CureM outcome unless explicitly labelled as a before-versus-after correction audit. The complete internal audit remains available separately; row-level identifiers are not reproduced here.

# Supplementary Table S1

Supplementary Table S1. Exposure clustering, missed-care threshold sensitivity, and CureM direction-harmonization audit.

**Panel A. Exposure clustering and within-nurse information**

| **Diagnostic** | **Value** |
| --- | --- |
| Observations in primary complete-case model | 480 |
| Nurse clusters | 196 |
| Singleton nurse clusters | 63 (32.1%) |
| Nurses with repeated observations | 133 |
| Repeated nurses with within-nurse variation in work rhythm/quantity | 123/133 |
| Repeated nurses without within-nurse variation in work rhythm/quantity | 10/133 |
| Nurse-level ICC for work rhythm/quantity | 0.491 |

**Panel B. Missed-care threshold sensitivity**

| **Scope** | **Outcome threshold** | **Predictor** | **OR (95% CI); p** | **Observations** | **Nurses** |
| --- | --- | --- | --- | --- | --- |
| Six hospitals, harmonized | 2-5 coded as missed | Work rhythm/quantity | OR 1.22 (1.05-1.43); p=.011 | 480 | 196 |
| Six hospitals, harmonized | 2-5 coded as missed | Assigned patients | OR 1.16 (0.98-1.38); p=.092 | 480 | 196 |
| Six hospitals, harmonized | 3-5 coded as missed | Work rhythm/quantity | OR 1.24 (1.03-1.49); p=.023 | 480 | 196 |
| Six hospitals, harmonized | 3-5 coded as missed | Assigned patients | OR 1.14 (0.94-1.40); p=.192 | 480 | 196 |
| Six hospitals, harmonized | 4-5 coded as missed | Work rhythm/quantity | OR 1.27 (0.99-1.64); p=.061 | 480 | 196 |
| Six hospitals, harmonized | 4-5 coded as missed | Assigned patients | OR 1.31 (1.01-1.70); p=.040 | 480 | 196 |
| Excluding Hospitals 4-5 | 2-5 coded as missed | Work rhythm/quantity | OR 1.18 (1.00-1.39); p=.055 | 420 | 171 |
| Excluding Hospitals 4-5 | 2-5 coded as missed | Assigned patients | OR 1.20 (1.00-1.44); p=.050 | 420 | 171 |
| Excluding Hospitals 4-5 | 3-5 coded as missed | Work rhythm/quantity | OR 1.26 (1.03-1.55); p=.022 | 420 | 171 |
| Excluding Hospitals 4-5 | 3-5 coded as missed | Assigned patients | OR 1.13 (0.92-1.39); p=.259 | 420 | 171 |
| Excluding Hospitals 4-5 | 4-5 coded as missed | Work rhythm/quantity | OR 1.40 (1.06-1.83); p=.016 | 420 | 171 |
| Excluding Hospitals 4-5 | 4-5 coded as missed | Assigned patients | OR 1.28 (0.96-1.71); p=.089 | 420 | 171 |

Panel C. Hospitals 4-5 CureM direction-harmonization audit

| **Hospital** | **Scenario** | **Mean missed care** | **L1 distance** | **Item-profile rho** | **Shifts with >=90% missed (%)** | **Hospital OR, robust GLM** | **Mean mental workload** |
| --- | --- | --- | --- | --- | --- | --- | --- |
| 4 | Original CureM direction | 33.8 | 1.60 | -0.29 | 92.1 | OR 77.12 (13.96-426.11) | 28.1 |
| 4 | Corrected CureM direction | 4.5 | 0.26 | 0.61 | 5.3 | OR 0.91 (0.30-2.75) | 28.1 |
| 5 | Original CureM direction | 32.9 | 1.53 | -0.62 | 80.5 | OR 66.49 (20.74-213.21) | 43.7 |
| 5 | Corrected CureM direction | 5.6 | 0.11 | 0.68 | 0.0 | OR 1.54 (0.62-3.81) | 43.7 |

Correction summary: the cluster-robust grouped-binomial GLM work rhythm/quantity OR changed from 1.32 before correction to 1.29 after correction; the primary exchangeable GEE OR changed from 1.28 to 1.24. The hospital-level correlation between mean work rhythm/quantity and mean missed care changed from r=-0.55 to r=+0.89. These diagnostics support correction of the CureM block only and do not support reversing workload items.

# Supplementary Table S2

**Supplementary Table S2. Missed-care domain definitions and item allocation.**

| **Domain** | **Number of items** | **Items** |
| --- | --- | --- |
| Fundamental mobility/self-care | 9 | CureM4; CureM5; CureM6; CureM7; CureM8; CureM9; CureM10; CureM11; CureM20 |
| Surveillance, safety and treatment | 14 | CureM12; CureM13; CureM14; CureM15; CureM16; CureM17; CureM18; CureM19; CureM21; CureM28; CureM29; CureM30; CureM31; CureM32 |
| Communication, education and relationship | 6 | CureM22; CureM23; CureM24; CureM25; CureM26; CureM27 |
| Planning, coordination and continuity | 8 | CureM1; CureM2; CureM3; CureM33; CureM34; CureM35; CureM36; CureM37 |

# Supplementary Table S3

**Supplementary Table S3. Missingness and complete-case selection.**

**Panel A. Complete-case exclusion reasons by variable**

| **Variable** | **Records missing total** | **Records missing among excluded** | **Nurses affected** | **Hospitals affected** | **Hospital counts** |
| --- | --- | --- | --- | --- | --- |
| nurse | 1 | 1 | 0 | 5 | 1=0; 2=0; 3=0; 4=0; 5=1; 6=0 |
| gender | 22 | 22 | 17 | 3; 5 | 1=0; 2=0; 3=3; 4=0; 5=19; 6=0 |
| eta_z | 22 | 22 | 17 | 3; 5 | 1=0; 2=0; 3=3; 4=0; 5=19; 6=0 |
| seniority_months_z | 19 | 19 | 14 | 5 | 1=0; 2=0; 3=0; 4=0; 5=19; 6=0 |
| workload_pace_z | 1 | 1 | 1 | 3 | 1=0; 2=0; 3=1; 4=0; 5=0; 6=0 |
| workload_mental_z | 1 | 1 | 1 | 3 | 1=0; 2=0; 3=1; 4=0; 5=0; 6=0 |
| workload_emotional_z | 1 | 1 | 1 | 3 | 1=0; 2=0; 3=1; 4=0; 5=0; 6=0 |
| workload_organization_z | 1 | 1 | 1 | 3 | 1=0; 2=0; 3=1; 4=0; 5=0; 6=0 |

**Panel B. Included versus excluded records**

| **Included in primary model** | **Observations** | **Nurses** | **Mean missed care** | **Median missed care** | **Mean denominator** | **Mean work rhythm/quantity** | **Mean patients** |
| --- | --- | --- | --- | --- | --- | --- | --- |
| Excluded | 22 | 17 | 6.0 | 4.5 | 36.0 | 41.5 | 5.6 |
| Included | 480 | 196 | 6.5 | 3.0 | 36.5 | 43.0 | 9.0 |

**Panel C. Primary model inclusion by hospital**

| **Hospital** | **Total observations** | **Included** | **Excluded** | **Nurses total** | **Nurses included** |
| --- | --- | --- | --- | --- | --- |
| 1 | 205 | 205 | 0 | 73 | 73 |
| 2 | 60 | 60 | 0 | 19 | 19 |
| 3 | 69 | 66 | 3 | 48 | 45 |
| 4 | 38 | 38 | 0 | 15 | 15 |
| 5 | 41 | 22 | 19 | 24 | 10 |
| 6 | 89 | 89 | 0 | 34 | 34 |

# Supplementary Table S4

Supplementary Table S4. Hospital-level distributions after missed care direction harmonization and denominator audit.

**Panel A. Hospital-level outcome and workload distributions**

| **Hospital** | **Observations** | **Nurses** | **Mean missed care** | **Median missed care** | **Zero missed care (%)** | **Mean mental workload** | **Mean work rhythm/quantity** |
| --- | --- | --- | --- | --- | --- | --- | --- |
| 1 | 205 | 73 | 5.2 | 2.0 | 32.2 | 90.7 | 41.2 |
| 2 | 60 | 19 | 10.8 | 9.0 | 13.3 | 89.4 | 60.7 |
| 3 | 69 | 48 | 6.3 | 2.0 | 39.1 | 80.6 | 46.0 |
| 4 | 38 | 15 | 4.5 | 1.0 | 44.7 | 28.1 | 29.4 |
| 5 | 41 | 24 | 5.6 | 3.0 | 19.5 | 43.7 | 39.3 |
| 6 | 89 | 34 | 7.8 | 5.0 | 27.0 | 78.4 | 40.2 |

**Panel B. Denominator audit: not-applicable versus missing item responses**

| **Scope** | **Hospital** | **Rows** | **Item slots** | **Applicable item responses** | **Not-applicable item responses** | **Missing item responses** | **Rows with denominator <37** | **Only not-applicable** | **Only missing** | **Both** |
| --- | --- | --- | --- | --- | --- | --- | --- | --- | --- | --- |
| overall | NA | 502 | 18574 | 18311 | 253 | 10 | 87 | 80 | 4 | 3 |
| by_hospital | 1 | 205 | 7585 | 7512 | 73 | 0 | 27 | 27 | 0 | 0 |
| by_hospital | 2 | 60 | 2220 | 2206 | 14 | 0 | 6 | 6 | 0 | 0 |
| by_hospital | 3 | 69 | 2553 | 2495 | 49 | 9 | 18 | 12 | 4 | 2 |
| by_hospital | 4 | 38 | 1406 | 1377 | 28 | 1 | 10 | 9 | 0 | 1 |
| by_hospital | 5 | 41 | 1517 | 1463 | 54 | 0 | 16 | 16 | 0 | 0 |
| by_hospital | 6 | 89 | 3293 | 3258 | 35 | 0 | 10 | 10 | 0 | 0 |

Supplementary Figure S1. Pooled CureM response distributions before and after direction harmonization. The original Hospital 4-5 profiles were reflected relative to Hospitals 1, 2, 3, and 6; recoding only CureM values 1-5 as 6-x aligned their distributions with the reference profile. Workload items were not recoded.

# Supplementary Table S5

**Supplementary Table S5. GEE model diagnostics.**

| **Model** | **Outcome grain** | **Working correlation** | **Rows** | **Shift records** | **Nurse clusters** | **Median cluster size** | **Max cluster size** | **Singleton clusters** | **Estimated alpha** |
| --- | --- | --- | --- | --- | --- | --- | --- | --- | --- |
| Primary GEE, hospital fixed effects | shift_grouped_binomial | exchangeable | 480 | 480 | 196 | 2.0 | 8 | 63 | 0.185 |
| Sensitivity: no age/gender/seniority | shift_grouped_binomial | exchangeable | 499 | 499 | 211 | 2.0 | 8 | 75 | 0.220 |
| Sensitivity: working independence | shift_grouped_binomial | independence | 480 | 480 | 196 | 2.0 | 8 | 63 | - |
| Ward fixed effects (quasi-separation) | shift_grouped_binomial | exchangeable | 480 | 480 | 196 | 2.0 | 8 | 63 | 0.598 |
| Sensitivity: excluding Hospitals 4-5 | shift_grouped_binomial | exchangeable | 420 | 420 | 171 | 2.0 | 8 | 58 | 0.147 |
| Within-between GEE | shift_grouped_binomial | exchangeable | 480 | 480 | 196 | 2.0 | 8 | 63 | 0.181 |
| Within-between, working independence | shift_grouped_binomial | independence | 480 | 480 | 196 | 2.0 | 8 | 63 | - |
| Within-between, excluding Hospitals 4-5 | shift_grouped_binomial | exchangeable | 420 | 420 | 171 | 2.0 | 8 | 58 | 0.143 |
| Item-level GEE, exchangeable | item_level_binary | exchangeable | 17520 | 480 | 196 | 74.0 | 295 | 63 | 0.250 |
| Item-level GEE, independence | item_level_binary | independence | 17520 | 480 | 196 | 74.0 | 295 | 63 | - |

# Supplementary Table S6

Supplementary Table S6. Primary grouped-binomial GEE and shift-level sensitivity analyses after CureM direction harmonization.

| **Model** | **Predictor** | **OR (95% CI)** | **p value** | **Observations** | **Nurses** | **Alpha** |
| --- | --- | --- | --- | --- | --- | --- |
| Primary GEE, hospital fixed effects | Work rhythm/quantity | OR 1.24 (1.03-1.49) | .023 | 480 | 196 | 0.185 |
| Primary GEE, hospital fixed effects | Mental workload | OR 0.94 (0.78-1.12) | .475 | 480 | 196 | 0.185 |
| Primary GEE, hospital fixed effects | Emotional workload | OR 1.12 (0.95-1.32) | .174 | 480 | 196 | 0.185 |
| Primary GEE, hospital fixed effects | Work organization | OR 1.00 (0.83-1.19) | .964 | 480 | 196 | 0.185 |
| Primary GEE, hospital fixed effects | Assigned patients | OR 1.14 (0.94-1.40) | .192 | 480 | 196 | 0.185 |
| Primary GEE, hospital fixed effects | Isolated patients | OR 0.92 (0.82-1.04) | .166 | 480 | 196 | 0.185 |
| Primary GEE, hospital fixed effects | Specialist-care patients | OR 1.04 (0.88-1.22) | .637 | 480 | 196 | 0.185 |
| No age/gender/seniority | Work rhythm/quantity | OR 1.24 (1.03-1.48) | .021 | 499 | 211 | 0.220 |
| No age/gender/seniority | Mental workload | OR 0.93 (0.77-1.11) | .417 | 499 | 211 | 0.220 |
| No age/gender/seniority | Emotional workload | OR 1.12 (0.95-1.31) | .170 | 499 | 211 | 0.220 |
| No age/gender/seniority | Work organization | OR 0.97 (0.81-1.15) | .706 | 499 | 211 | 0.220 |
| No age/gender/seniority | Assigned patients | OR 1.17 (0.96-1.43) | .127 | 499 | 211 | 0.220 |
| No age/gender/seniority | Isolated patients | OR 0.92 (0.82-1.03) | .130 | 499 | 211 | 0.220 |
| No age/gender/seniority | Specialist-care patients | OR 1.03 (0.89-1.19) | .705 | 499 | 211 | 0.220 |
| Working independence | Work rhythm/quantity | OR 1.29 (1.07-1.56) | .009 | 480 | 196 | - |
| Working independence | Mental workload | OR 0.93 (0.76-1.13) | .452 | 480 | 196 | - |
| Working independence | Emotional workload | OR 1.11 (0.94-1.31) | .227 | 480 | 196 | - |
| Working independence | Work organization | OR 0.97 (0.81-1.17) | .767 | 480 | 196 | - |
| Working independence | Assigned patients | OR 1.18 (0.96-1.45) | .121 | 480 | 196 | - |
| Working independence | Isolated patients | OR 0.91 (0.79-1.04) | .161 | 480 | 196 | - |
| Working independence | Specialist-care patients | OR 1.06 (0.90-1.26) | .481 | 480 | 196 | - |
| Ward fixed effects | Work rhythm/quantity | Not estimable (quasi-separation) | - | 480 | 196 | 0.598 |
| Ward fixed effects | Mental workload | Not estimable (quasi-separation) | - | 480 | 196 | 0.598 |
| Ward fixed effects | Emotional workload | Not estimable (quasi-separation) | - | 480 | 196 | 0.598 |
| Ward fixed effects | Work organization | Not estimable (quasi-separation) | - | 480 | 196 | 0.598 |
| Ward fixed effects | Assigned patients | Not estimable (quasi-separation) | - | 480 | 196 | 0.598 |
| Ward fixed effects | Isolated patients | Not estimable (quasi-separation) | - | 480 | 196 | 0.598 |
| Ward fixed effects | Specialist-care patients | Not estimable (quasi-separation) | - | 480 | 196 | 0.598 |
| Excluding Hospitals 4-5 | Work rhythm/quantity | OR 1.26 (1.03-1.55) | .022 | 420 | 171 | 0.147 |
| Excluding Hospitals 4-5 | Mental workload | OR 0.91 (0.74-1.12) | .382 | 420 | 171 | 0.147 |
| Excluding Hospitals 4-5 | Emotional workload | OR 1.09 (0.92-1.29) | .323 | 420 | 171 | 0.147 |
| Excluding Hospitals 4-5 | Work organization | OR 0.99 (0.82-1.20) | .938 | 420 | 171 | 0.147 |
| Excluding Hospitals 4-5 | Assigned patients | OR 1.13 (0.92-1.39) | .259 | 420 | 171 | 0.147 |
| Excluding Hospitals 4-5 | Isolated patients | OR 0.93 (0.84-1.04) | .210 | 420 | 171 | 0.147 |
| Excluding Hospitals 4-5 | Specialist-care patients | OR 1.08 (0.92-1.27) | .342 | 420 | 171 | 0.147 |

Supplementary Figure S2. Work rhythm/quantity odds ratios across estimable shift-level grouped-binomial GEE sensitivity models after CureM direction harmonization. The ward fixed-effects GEE is omitted because quasi-separation made its coefficients non-estimable.

# Supplementary Table S7

**Supplementary Table S7. Within-between grouped-binomial GEE model.**

**Panel A. Primary within–between analysis including all complete-case observations**

| **Model** | **Predictor** | **OR (95% CI)** | **p value** | **Observations** | **Nurses** |
| --- | --- | --- | --- | --- | --- |
| Within-between GEE, hospital fixed effects | Work rhythm/quantity: within-nurse | OR 1.03 (0.90-1.17) | .649 | 480 | 196 |
| Within-between GEE, hospital fixed effects | Mental workload: within-nurse | OR 1.02 (0.94-1.10) | .664 | 480 | 196 |
| Within-between GEE, hospital fixed effects | Emotional workload: within-nurse | OR 1.05 (0.95-1.16) | .320 | 480 | 196 |
| Within-between GEE, hospital fixed effects | Work organization: within-nurse | OR 1.04 (0.92-1.18) | .538 | 480 | 196 |
| Within-between GEE, hospital fixed effects | Assigned patients: within-nurse | OR 1.02 (0.90-1.15) | .756 | 480 | 196 |
| Within-between GEE, hospital fixed effects | Isolated patients: within-nurse | OR 0.99 (0.92-1.07) | .864 | 480 | 196 |
| Within-between GEE, hospital fixed effects | Specialist-care patients: within-nurse | OR 0.99 (0.90-1.09) | .852 | 480 | 196 |
| Within-between GEE, hospital fixed effects | Work rhythm/quantity: between-nurse | OR 1.42 (1.11-1.82) | .005 | 480 | 196 |
| Within-between GEE, hospital fixed effects | Mental workload: between-nurse | OR 0.89 (0.70-1.12) | .317 | 480 | 196 |
| Within-between GEE, hospital fixed effects | Emotional workload: between-nurse | OR 1.12 (0.92-1.35) | .255 | 480 | 196 |
| Within-between GEE, hospital fixed effects | Work organization: between-nurse | OR 0.88 (0.68-1.13) | .318 | 480 | 196 |
| Within-between GEE, hospital fixed effects | Assigned patients: between-nurse | OR 1.18 (0.92-1.51) | .192 | 480 | 196 |
| Within-between GEE, hospital fixed effects | Isolated patients: between-nurse | OR 0.87 (0.71-1.06) | .154 | 480 | 196 |
| Within-between GEE, hospital fixed effects | Specialist-care patients: between-nurse | OR 1.07 (0.88-1.29) | .494 | 480 | 196 |

**Panel B. Sensitivity analysis restricted to nurses with at least two complete-case shift observations**

| Model | Predictor | OR (95% CI) | p value | Observations | Nurses |
| --- | --- | --- | --- | --- | --- |
| Restricted within–between GEE, hospital fixed effects | Work rhythm/quantity: within-nurse | 1.03 (0.90–1.18) | .646 | 417 | 133 |
| Restricted within–between GEE, hospital fixed effects | Mental workload: within-nurse | 1.02 (0.94–1.11) | .634 | 417 | 133 |
| Restricted within–between GEE, hospital fixed effects | Emotional workload: within-nurse | 1.05 (0.95–1.17) | .328 | 417 | 133 |
| Restricted within–between GEE, hospital fixed effects | Work organization: within-nurse | 1.04 (0.91–1.19) | .544 | 417 | 133 |
| Restricted within–between GEE, hospital fixed effects | Assigned patients: within-nurse | 1.02 (0.89–1.16) | .772 | 417 | 133 |
| Restricted within–between GEE, hospital fixed effects | Isolated patients: within-nurse | 0.99 (0.91–1.08) | .857 | 417 | 133 |
| Restricted within–between GEE, hospital fixed effects | Specialist-care patients: within-nurse | 0.99 (0.90–1.09) | .840 | 417 | 133 |
| Restricted within–between GEE, hospital fixed effects | Work rhythm/quantity: between-nurse | 1.32 (1.00–1.75) | .054 | 417 | 133 |
| Restricted within–between GEE, hospital fixed effects | Mental workload: between-nurse | 0.90 (0.70–1.16) | .422 | 417 | 133 |
| Restricted within–between GEE, hospital fixed effects | Emotional workload: between-nurse | 1.04 (0.85–1.28) | .688 | 417 | 133 |
| Restricted within–between GEE, hospital fixed effects | Work organization: between-nurse | 1.03 (0.78–1.36) | .842 | 417 | 133 |
| Restricted within–between GEE, hospital fixed effects | Assigned patients: between-nurse | 1.46 (1.05–2.01) | .023 | 417 | 133 |
| Restricted within–between GEE, hospital fixed effects | Isolated patients: between-nurse | 0.87 (0.62–1.20) | .384 | 417 | 133 |
| Restricted within–between GEE, hospital fixed effects | Specialist-care patients: between-nurse | 1.08 (0.88–1.34) | .460 | 417 | 133 |

*Note.* OR = odds ratio; CI = confidence interval; GEE = generalized estimating equations. The model was restricted to nurses contributing at least two complete-case shift observations and included all 14 within- and between-nurse workload components, age, sex, professional seniority, shift, and hospital fixed effects. Continuous predictors were standardized. The model included 417 shift records from 133 nurses; the estimated exchangeable working correlation was 0.173. Reported p values are nominal. Benjamini–Hochberg q values were calculated across the 14 focal workload coefficients; none remained significant at q < .05. The q value was .320 for the between-nurse assigned-patient coefficient and .376 for the between-nurse work rhythm/quantity coefficient; all remaining q values were .857.

# Supplementary Table S8

**Supplementary Table S8. Item-level GEE sensitivity models.**

| **Model** | **Predictor** | **OR (95% CI)** | **p value** | **Item rows** | **Shift records** | **Nurses** | **Alpha** |
| --- | --- | --- | --- | --- | --- | --- | --- |
| Item-level GEE, exchangeable | Work rhythm/quantity | OR 1.06 (0.83-1.35) | .629 | 17520 | 480 | 196 | 0.250 |
| Item-level GEE, exchangeable | Mental workload | OR 1.04 (0.84-1.29) | .703 | 17520 | 480 | 196 | 0.250 |
| Item-level GEE, exchangeable | Emotional workload | OR 1.20 (0.93-1.55) | .153 | 17520 | 480 | 196 | 0.250 |
| Item-level GEE, exchangeable | Work organization | OR 1.09 (0.87-1.37) | .460 | 17520 | 480 | 196 | 0.250 |
| Item-level GEE, exchangeable | Assigned patients | OR 1.04 (0.75-1.45) | .811 | 17520 | 480 | 196 | 0.250 |
| Item-level GEE, exchangeable | Isolated patients | OR 0.96 (0.86-1.08) | .517 | 17520 | 480 | 196 | 0.250 |
| Item-level GEE, exchangeable | Specialist-care patients | OR 0.99 (0.81-1.20) | .903 | 17520 | 480 | 196 | 0.250 |
| Item-level GEE, working independence | Work rhythm/quantity | OR 1.31 (1.07-1.60) | .009 | 17520 | 480 | 196 | - |
| Item-level GEE, working independence | Mental workload | OR 0.92 (0.75-1.14) | .455 | 17520 | 480 | 196 | - |
| Item-level GEE, working independence | Emotional workload | OR 1.12 (0.94-1.33) | .220 | 17520 | 480 | 196 | - |
| Item-level GEE, working independence | Work organization | OR 0.97 (0.80-1.18) | .760 | 17520 | 480 | 196 | - |
| Item-level GEE, working independence | Assigned patients | OR 1.19 (0.95-1.48) | .125 | 17520 | 480 | 196 | - |
| Item-level GEE, working independence | Isolated patients | OR 0.90 (0.78-1.04) | .157 | 17520 | 480 | 196 | - |
| Item-level GEE, working independence | Specialist-care patients | OR 1.07 (0.89-1.28) | .480 | 17520 | 480 | 196 | - |

# Supplementary Table S9

**Supplementary Table S9. Domain-specific grouped-binomial GEE models.**

| **Domain** | **Predictor** | **OR (95% CI)** | **p value** | **q value, 28 tests** | **q value, pace family** | **Observations** | **Nurses** |
| --- | --- | --- | --- | --- | --- | --- | --- |
| Fundamental mobility/self-care | Work rhythm/quantity | OR 1.11 (0.91-1.37) | .309 | .617 | .309 | 480 | 196 |
| Fundamental mobility/self-care | Mental workload | OR 0.95 (0.77-1.18) | .669 | .872 | - | 480 | 196 |
| Fundamental mobility/self-care | Emotional workload | OR 1.13 (0.94-1.36) | .198 | .500 | - | 480 | 196 |
| Fundamental mobility/self-care | Work organization | OR 1.11 (0.90-1.36) | .338 | .630 | - | 480 | 196 |
| Fundamental mobility/self-care | Assigned patients | OR 1.15 (0.92-1.45) | .224 | .500 | - | 480 | 196 |
| Fundamental mobility/self-care | Isolated patients | OR 0.99 (0.86-1.14) | .912 | .982 | - | 480 | 196 |
| Fundamental mobility/self-care | Specialist-care patients | OR 1.02 (0.85-1.21) | .859 | .962 | - | 480 | 196 |
| Surveillance, safety and treatment | Work rhythm/quantity | OR 1.26 (1.00-1.59) | .049 | .458 | .065 | 480 | 196 |
| Surveillance, safety and treatment | Mental workload | OR 0.89 (0.73-1.08) | .232 | .500 | - | 480 | 196 |
| Surveillance, safety and treatment | Emotional workload | OR 1.15 (0.96-1.38) | .139 | .488 | - | 480 | 196 |
| Surveillance, safety and treatment | Work organization | OR 0.95 (0.78-1.16) | .643 | .872 | - | 480 | 196 |
| Surveillance, safety and treatment | Assigned patients | OR 1.25 (0.98-1.59) | .069 | .463 | - | 480 | 196 |
| Surveillance, safety and treatment | Isolated patients | OR 0.89 (0.76-1.03) | .115 | .488 | - | 480 | 196 |
| Surveillance, safety and treatment | Specialist-care patients | OR 1.09 (0.90-1.33) | .370 | .648 | - | 480 | 196 |
| Communication, education and relationship | Work rhythm/quantity | OR 1.38 (1.08-1.78) | .011 | .205 | .029 | 479 | 196 |
| Communication, education and relationship | Mental workload | OR 1.00 (0.74-1.34) | .986 | .986 | - | 479 | 196 |
| Communication, education and relationship | Emotional workload | OR 1.04 (0.83-1.31) | .712 | .872 | - | 479 | 196 |
| Communication, education and relationship | Work organization | OR 0.95 (0.74-1.23) | .716 | .872 | - | 479 | 196 |
| Communication, education and relationship | Assigned patients | OR 1.00 (0.78-1.28) | .981 | .986 | - | 479 | 196 |
| Communication, education and relationship | Isolated patients | OR 0.88 (0.72-1.06) | .166 | .500 | - | 479 | 196 |
| Communication, education and relationship | Specialist-care patients | OR 1.09 (0.87-1.36) | .456 | .709 | - | 479 | 196 |
| Planning, coordination and continuity | Work rhythm/quantity | OR 1.33 (1.06-1.67) | .015 | .205 | .029 | 480 | 196 |
| Planning, coordination and continuity | Mental workload | OR 0.92 (0.73-1.15) | .448 | .709 | - | 480 | 196 |
| Planning, coordination and continuity | Emotional workload | OR 1.16 (0.96-1.39) | .124 | .488 | - | 480 | 196 |
| Planning, coordination and continuity | Work organization | OR 0.94 (0.77-1.14) | .525 | .774 | - | 480 | 196 |
| Planning, coordination and continuity | Assigned patients | OR 1.16 (0.93-1.44) | .185 | .500 | - | 480 | 196 |
| Planning, coordination and continuity | Isolated patients | OR 0.87 (0.75-1.02) | .083 | .463 | - | 480 | 196 |
| Planning, coordination and continuity | Specialist-care patients | OR 0.97 (0.80-1.18) | .771 | .900 | - | 480 | 196 |

# Supplementary Table S10

**Supplementary Table S10. Common support for exploratory objective workload categories.**

| **Hospital** | **Variable** | **Category** | **Observations** | **Mean missed care** |
| --- | --- | --- | --- | --- |
| 1 | n_patients_class | patients_le_7 | 46 | 3.6 |
| 1 | n_patients_class | patients_8_10 | 136 | 5.4 |
| 1 | n_patients_class | patients_ge_11 | 23 | 7.0 |
| 2 | n_patients_class | patients_le_7 | 10 | 7.3 |
| 2 | n_patients_class | patients_8_10 | 48 | 11.5 |
| 2 | n_patients_class | patients_ge_11 | 2 | 11.0 |
| 3 | n_patients_class | patients_le_7 | 15 | 10.2 |
| 3 | n_patients_class | patients_8_10 | 36 | 5.1 |
| 3 | n_patients_class | patients_ge_11 | 18 | 5.4 |
| 4 | n_patients_class | patients_le_7 | 30 | 4.0 |
| 4 | n_patients_class | patients_8_10 | 7 | 7.0 |
| 4 | n_patients_class | patients_ge_11 | 1 | 0.0 |
| 5 | n_patients_class | patients_le_7 | 40 | 5.2 |
| 5 | n_patients_class | patients_8_10 | 1 | 24.0 |
| 6 | n_patients_class | patients_le_7 | 1 | 1.0 |
| 6 | n_patients_class | patients_8_10 | 36 | 4.2 |
| 6 | n_patients_class | patients_ge_11 | 52 | 10.4 |
| 1 | n_isolated_class | isolated_0 | 97 | 5.0 |
| 1 | n_isolated_class | isolated_1_2 | 95 | 5.1 |
| 1 | n_isolated_class | isolated_ge_3 | 13 | 7.2 |
| 2 | n_isolated_class | isolated_0 | 44 | 11.2 |
| 2 | n_isolated_class | isolated_1_2 | 10 | 9.8 |
| 2 | n_isolated_class | isolated_ge_3 | 6 | 9.7 |
| 3 | n_isolated_class | isolated_0 | 51 | 7.3 |
| 3 | n_isolated_class | isolated_1_2 | 12 | 2.0 |
| 3 | n_isolated_class | isolated_ge_3 | 6 | 6.0 |
| 4 | n_isolated_class | isolated_0 | 37 | 4.6 |
| 4 | n_isolated_class | isolated_1_2 | 1 | 0.0 |
| 5 | n_isolated_class | isolated_0 | 3 | 2.3 |
| 5 | n_isolated_class | isolated_1_2 | 22 | 6.5 |
| 5 | n_isolated_class | isolated_ge_3 | 16 | 4.9 |
| 6 | n_isolated_class | isolated_0 | 74 | 7.5 |
| 6 | n_isolated_class | isolated_1_2 | 12 | 10.5 |
| 6 | n_isolated_class | isolated_ge_3 | 3 | 4.3 |
| 1 | n_special_needs_class | special_1 | 40 | 5.7 |
| 1 | n_special_needs_class | special_2 | 69 | 4.8 |
| 1 | n_special_needs_class | special_ge_3 | 96 | 5.3 |
| 2 | n_special_needs_class | special_1 | 20 | 6.8 |
| 2 | n_special_needs_class | special_2 | 1 | 13.0 |
| 2 | n_special_needs_class | special_ge_3 | 39 | 12.8 |
| 3 | n_special_needs_class | special_1 | 20 | 7.0 |
| 3 | n_special_needs_class | special_2 | 32 | 7.2 |
| 3 | n_special_needs_class | special_ge_3 | 17 | 3.6 |
| 4 | n_special_needs_class | special_1 | 23 | 6.5 |
| 4 | n_special_needs_class | special_2 | 6 | 3.5 |
| 4 | n_special_needs_class | special_ge_3 | 9 | 0.0 |
| 5 | n_special_needs_class | special_1 | 6 | 3.8 |
| 5 | n_special_needs_class | special_2 | 32 | 5.9 |
| 5 | n_special_needs_class | special_ge_3 | 3 | 6.3 |
| 6 | n_special_needs_class | special_1 | 32 | 5.0 |
| 6 | n_special_needs_class | special_2 | 24 | 8.6 |
| 6 | n_special_needs_class | special_ge_3 | 33 | 9.9 |
| 1 | complexity_class | complexity_0_2 | 91 | 5.7 |
| 1 | complexity_class | complexity_3 | 79 | 4.4 |
| 1 | complexity_class | complexity_4 | 35 | 5.8 |
| 2 | complexity_class | complexity_0_2 | 11 | 4.4 |
| 2 | complexity_class | complexity_3 | 25 | 12.0 |
| 2 | complexity_class | complexity_4 | 24 | 12.6 |
| 3 | complexity_class | complexity_0_2 | 17 | 4.9 |
| 3 | complexity_class | complexity_3 | 25 | 5.7 |
| 3 | complexity_class | complexity_4 | 27 | 7.7 |
| 4 | complexity_class | complexity_0_2 | 20 | 3.4 |
| 4 | complexity_class | complexity_3 | 15 | 6.5 |
| 4 | complexity_class | complexity_4 | 3 | 2.0 |
| 5 | complexity_class | complexity_0_2 | 18 | 2.7 |
| 5 | complexity_class | complexity_3 | 21 | 8.1 |
| 5 | complexity_class | complexity_4 | 2 | 5.5 |
| 6 | complexity_class | complexity_0_2 | 36 | 6.6 |
| 6 | complexity_class | complexity_3 | 37 | 8.1 |
| 6 | complexity_class | complexity_4 | 16 | 9.6 |

# Supplementary Table S11

**Supplementary Table S11. Patient complexity and perceived workload dimensions.**

| **Outcome** | **Predictor** | **Standardized beta** | **95% CI** | **p value** | **Observations** | **Adjusted R-squared** |
| --- | --- | --- | --- | --- | --- | --- |
| workload_pace | Patient complexity | 0.45 | 0.33-0.56 | <.001 | 480 | 0.326 |
| workload_mental | Patient complexity | 0.13 | 0.06-0.21 | <.001 | 480 | 0.513 |
| workload_emotional | Patient complexity | 0.28 | 0.16-0.41 | <.001 | 480 | 0.119 |
| workload_organization | Patient complexity | 0.35 | 0.26-0.44 | <.001 | 480 | 0.240 |
